# Analysis of Drug Metabolizing Gene Panel in Osteosarcoma Patients Identifies Association Between Variants in *SULT1E1, CYP2B6* and *CYP4F8* and Methotrexate Levels and Toxicities

**DOI:** 10.3389/fphar.2020.01241

**Published:** 2020-08-12

**Authors:** Evelien G. E. Hurkmans, Marije J. Klumpers, Sita H. Vermeulen, Melanie M. Hagleitner, Uta Flucke, H. W. Bart Schreuder, Hans Gelderblom, Johannes Bras, Henk-Jan Guchelaar, Marieke J. H. Coenen, D. Maroeska W. M. te Loo

**Affiliations:** ^1^ Department of Human Genetics, Radboud Institute for Health Sciences, Radboud University Medical Center, Nijmegen, Netherlands; ^2^ Department of Pediatrics, Radboud Institute for Molecular Life Sciences, Radboud University Medical Center, Nijmegen, Netherlands; ^3^ Department for Health Evidence, Radboud Institute for Health Sciences, Radboud University Medical Center, Nijmegen, Netherlands; ^4^ Princess Máxima Center for Pediatric Oncology, Utrecht, Netherlands; ^5^ Department of Pathology, Radboud University Medical Center, Nijmegen, Netherlands; ^6^ Department of Orthopedic Surgery, Radboud University Medical Center, Nijmegen, Netherlands; ^7^ Department of Medical Oncology, Leiden University Medical Center, Leiden, Netherlands; ^8^ Department of Pathology, Academic Medical Center, Amsterdam, Netherlands; ^9^ Department of Clinical Pharmacy & Toxicology, Leiden University Medical Center, Leiden, Netherlands; ^10^ Department of Pediatrics, Radboud Institute for Health Sciences, Radboud University Medical Center, Nijmegen, Netherlands

**Keywords:** methotrexate, osteosarcoma, pharmacogenetics, absorption, distribution, metabolism, and excretion (ADME), plasma levels, toxicity

## Abstract

High-dose methotrexate is a cornerstone agent in the chemotherapeutic treatment of patients with osteosarcoma. However, patients often develop methotrexate-induced toxicities. We aim to identify determinants of methotrexate-induced toxicities in osteosarcoma patients by investigating the relation between drug plasma levels, methotrexate-induced toxicities, and germline variants in genes related to drug absorption, distribution, metabolism, and elimination. A cohort of 114 osteosarcoma patients was genotyped for 1,931 variants in 231 genes using the Drug Metabolism Enzymes and Transporters Plus array. Methotrexate plasma levels and laboratory measurements during and after high-dose methotrexate treatment concerning renal function, liver damage, and myelopoiesis to reflect toxicity outcomes were obtained. One hundred and thirteen patients and a subset of 545 variants in 176 genes passed quality control checks. Methotrexate plasma levels showed associations with creatinine, alanine aminotransferase, and hemoglobin. Genetic variant rs3736599 in the 5’-untranslated region of *SULT1E1* was associated with lower 48 hour methotrexate plasma levels [coef -0.313 (95% CI -0.459 – -0.167); p = 2.60 × 10^-5^]. Association with methotrexate-induced decreased thrombocyte counts was found for two intronic variants in *CYP2B6* {rs4803418 [coef -0.187 (95% CI -0.275 – -0.099); p = 3.04 × 10^-5^] and rs4803419 [coef -0.186 (95% CI -0.278 – -0.093); p = 8.80 × 10^-5^]}. An association with increased thrombocyte counts was identified for the intronic variant rs4808326 in *CYP4F8* [coef 0.193 (95% CI 0.099 – 0.287); p = 6.02 × 10^-5^]. Moreover, a secondary analysis with a binary approach using CTCAE toxicity criteria resulted in a nominal significant associations (p < 0.05) for two out of three variants (rs4803418 and rs4808326). This is the first study to identify genetic variants in *SULT1E1, CYP2B6*, and *CYP4F8* to be associated with methotrexate pharmacokinetics and toxicities. Validation of these variants in an independent cohort and further functional investigation of variants in the identified genes is needed to determine if and how they affect methotrexate plasma levels and the development of methotrexate-induced toxicities.

## Introduction

Methotrexate (MTX) is an antifolate agent widely used in oncologic treatment. In high doses (≥1 g/m²), MTX is used as a cornerstone agent in the chemotherapeutic treatment of osteosarcoma, the most common primary bone tumor in children and adolescents (Jolivet et al., 1983; Treon and Chabner, 1996). Introduction of chemotherapeutic treatment with a regimen of the agents doxorubicin, cisplatin and high dose (HD-)MTX in osteosarcoma has resulted in tremendous increase of patients’ survival rates compared to surgery alone (5-year overall survival up to 70% compared to <20%, respectively) (Link et al., 1986; Bacci et al., 1993; Hagleitner et al., 2012; Gatta et al., 2014). Despite its contribution to improved prognosis, HD-MTX treatment can however lead to harmful toxicities including renal toxicity, myelosuppression and liver damage (Jolivet et al., 1983; Rask et al., 1998; Csordas et al., 2013). These HD-MTX-related toxicities can occur despite appropriate leucovorin rescue, intensive hydration, and monitoring of drug plasma levels. Clinical factors, such as age and kidney function, are known to contribute to the risk of developing toxicities but do not sufficiently explain all interpatient variation (Csordas et al., 2013; Cheng et al., 2018). Pharmacogenetic studies aim to fill the gap of unexplained interpatient variability in drug response by investigating how genetic variants affect relevant traits such as drug plasma levels and drug-related adverse events.

Studies investigating the impact of genetic variants on MTX pharmacokinetics and toxicities have already shown significant results. A genome-wide association study (GWAS) in patients with acute lymphoblastic leukemia (ALL) showed that genetic variants in *SLCO1B1* are associated with MTX clearance (Trevino et al., 2009). This association has been replicated by others (Lopez-Lopez et al., 2011; Radtke et al., 2013; Ramsey et al., 2013; Csordas et al., 2014; Goricar et al., 2014; Zhang et al., 2014; Liu et al., 2017). Osteosarcoma patients receive a substantial higher cumulative dose of MTX compared to patients with ALL (12 g/m² compared to 2 g/m² in most treatment protocols, respectively). This is particularly relevant as important differences are described between high and lower MTX dosages concerning cellular transport pathways (Schmiegelow, 2009). Therefore, one needs to be precautious with direct generalization of pharmacogenetic associations found in other malignancies or other low dose MTX-treated traits such as autoimmune diseases to osteosarcoma. In osteosarcoma, only candidate gene studies have been performed to identify relevant pharmacogenetic variants, resulting in the identification of statistically significantly associations between variants in *MTHFR*, *MTR* and *ABCB1*, and MTX pharmacokinetics, toxicities and survival rates (Patino-Garcia et al., 2009; Windsor et al., 2012; Jabeen et al., 2015; Park and Shin, 2016; Lambrecht et al., 2017). Previously, our group investigated the role of a genetic variant in *MTHFR* (rs1801133) in MTX-induced liver toxicity in patients with osteosarcoma and ALL (Hagleitner et al., 2014). The present study follows up on that in a larger cohort, and looking beyond candidate genes by exploring a broad panel of variants in genes involved in drug absorption, distribution, metabolism, and excretion (ADME). This panel contained part of the previously identified genetic variants in MTX pathways, but also provided us with a broader and unbiased view on the contribution of ADME gene variation on variability in HD-MTX response. This study focused on MTX plasma levels as well as laboratory markers for HD-MTX-induced renal toxicity, liver damage, and bone marrow toxicity in patients with osteosarcoma.

## Materials and Methods

### Patients and Treatment

The study cohort consisted of 114 patients diagnosed with primary, high-grade osteosarcoma, who were treated between 2003 and 2014 at (pediatric) oncology departments of four Dutch hospitals (Radboud university medical center; Leiden University Medical Center; Academic Medical Center Amsterdam; University Medical Center Groningen). Inclusion criteria were: age ≤45 years, self-reported Caucasian ethnicity, and treatment according to the EURAMOS-1 protocol (Whelan et al., 2015). The current study was approved by the institutional review board of the Radboud university medical center (Commissie Mensgebonden Onderzoek Regio Arnhem Nijmegen), and approval for inclusion of patients in other institutes was obtained from institutional ethics committees. Written informed consent was acquired from all patients and/or their parents. According to the treatment protocol, patients received a maximum of 12 courses HD-MTX (12 g/m² per course) as a 4 h infusion, together with adequate hydration and urinary alkalinization. Leucovorin rescue (15 mg/m²) was started 24–28 h after start of MTX infusion and was prolonged if MTX plasma levels were >0.40 µmol/L at 48 h. In addition to HD-MTX, chemotherapeutic treatment consisted of doxorubicin (maximum cumulative dose: 450 mg/m²) and cisplatin (maximum cumulative dose: 480 mg/m²), with or without additional ifosfamide/etoposide or interferon-α depending on randomized EURAMOS-1 treatment arm. None of the patients received trimethoprim-sulfamethoxazole during treatment due to its interaction with MTX.

### MTX Plasma Levels and Toxicity Data

MTX plasma levels and laboratory results of MTX-induced toxicities after each course of MTX were retrospectively collected from electronic medical records. MTX plasma levels were routinely measured at 48 h after initiation of MTX infusion by a fluorescence polarization immunoassay (TDx/FLx, Abott Diagnostics, The Hague, The Netherlands) or enzyme immunoassay (for three patients) (Syva Emit TDM assay, Siemens Healthcare, Hoofddorp, The Netherlands), without differences in reference value. For renal toxicity, creatinine plasma levels at 48 h and after one week (ranging from day five until day nine) after start of MTX infusion were collected, in order to assess both the acute and later effects of MTX on renal cells. To analyze liver damage, plasma levels of alanine aminotransferase (ALAT) and aspartate aminotransferase (ASAT) at 48 h were obtained. For bone marrow toxicity, levels of hemoglobin and counts of leukocytes, thrombocytes and neutrophils were collected, approximately one week (ranging from day five until day nine) after MTX infusion.

### Genotyping

Germline DNA was isolated from blood (n = 54) using the QIAamp DNA Blood Midi kit (Qiagen, Venlo, The Netherlands) or from saliva (n = 53) using the Oragene saliva collection kit (DNA Genotek, Kanata, Ontario, Canada) according to the manufacturer’s protocols. For patients who had passed away before study inclusion (n = 7), germline DNA was isolated from normal formalin-fixed paraffin-embedded bone tissue as described previously (Vos et al., 2015). DNA samples were genotyped for 1,936 genetic variants across 231 genes involved in drug absorption, distribution, metabolism, and excretion, using the DMET Plus array (Affymetrix UK Ltd, High Wycombe, UK) according to the manufacturer’s instructions. Genotypes were determined with DMET console software version 1.3 (Affymetrix UK Ltd, High Wycombe, UK) using the Dynamic Genotype Boundaries algorithm version 2. After exclusion of copy number variants, X-chromosomal variants and tri-allelic variants present on the array, quality control (QC) was performed. Variants with unreliable cluster plots, i.e. plots without distinct cluster boundaries, were excluded. Further QC consisted of exclusion of variants with a call rate below 0.90, a minor allele frequency (MAF) below 0.05, and variants, which deviated from the Hardy Weinberg equilibrium (HWE p < 0.0001), and exclusion of samples with a call rate below 0.90.

### Statistical Analyses

#### Analyses With Continuous Outcomes

First, analyses using continuous outcomes for MTX levels and toxicities were performed. After assessing normality of the data, Spearman’s rank-order correlation (*r_s_*) was used to assess the correlation between MTX plasma levels and toxicity markers at different time points during treatment. Genetic association analyses for MTX plasma levels and toxicity markers were performed using Generalized Estimating Equation (GEE) analysis, an extended linear regression analysis in which correlations among repeated measurements obtained from individual subjects over time are taken into account. Sex, age (at diagnosis), and cumulative MTX dose were tested for association with MTX plasma levels and toxicity markers using GEE analysis. Variables that showed an association at p<0.05 were included as a covariate in multivariate genetic association analyses to reduce the variation explained by these covariates on the outcome variables, and thereby increase the precision of the estimate of the effects of the genetic variants. A description of included covariates in analysis of each toxicity marker is provided in the **Supplementary** **Tables S3–S10**. Creatinine plasma level one week after MTX infusion was included as a covariate in all association analyses (except in analyses of renal toxicity) to adjust for decreased renal clearance of MTX. Prior to the GEE analysis, MTX plasma levels, and levels of thrombocytes, leukocytes, neutrophils, ALAT and ASAT were log-transformed to obtain normal distributions. Association analyses were performed using family(gaussian), link(identity), correlation(exchangeable), and vce(robust) as options of the *xtgee* command in STATA. Regression coefficients and corresponding 95% confidence intervals were generated for each genetic variant under the assumption of an additive genetic model. Associations were considered statistically significant if they surpassed the Bonferroni-corrected p-value threshold (p = 0.05 divided by the number of variants including in the analyses after QC). Genetic association analyses were performed using STATA version 11.2 (Stata corporation, College Station, TX, USA), all other analyses using IBM SPSS Statistics version 22 (SPSS Inc., Chicago, Ill, USA).

#### Analyses With Binary Outcomes

To further assess the clinical relevance of genetic associations, additional analyses was performed treating the toxicity markers as binary (case-control) outcomes. Grading of laboratory results was performed according to the Common Terminology Criteria for Adverse Events (CTCAE) version 5.0 of the National Cancer Institute (**Supplementary Table S11**) (National Cancer Institute PROCSG, 2017). Reference values for the laboratory results according to the hospital of inclusion, were used as “upper limit of normal” and “lower limit of normal”. The values of the CTCAE grades for each toxicity outcome were evaluated to determine a clinical relevant cut-off for case-control designation. For the CTCAE terms “Alanine aminotransferase increased”, “Aspartate aminotransferase increased”, “White blood cell decreased”, and “Anemia”, grade 0 and 1 were considered controls and grade 2, 3, and 4 (and if applicable, grade 5) were considered cases. For “Neutrophil count decreased”, grade 0, 1, and 2 were assigned as controls and grade 3 and 4 as cases. For “Platelet count decreased”, patients with grade 0 were considered controls, and patients with grade 1, 2, 3, and 4 were considered cases. Performing an association analyses with CTCAE graded outcomes was not feasible for renal damage, as over 95% of all creatinine measurements were graded as grade 0 according to CTCAE term “Creatinine increased”. Logistic multivariate GEE analysis was performed, including covariates that showed an association (p < 0.05) with the graded toxicity endpoint (described in **Supplementary Tables S3–S10**). Also, it was performed using family(binomial), link(logit), correlation(exchangeable), and vce(robust) as options of the *xtgee* command in STATA. Other than that, the same approach was used as the analyses of continuous outcomes (as descripted above).

## Results

### Patient Characteristics

Characteristics of the included osteosarcoma patients are provided in **Table 1**. The patients received a total of 1,256 MTX courses. All patients were treated according to the EURAMOS-1 protocol. 42.0% of the patients received a chemotherapeutic regimen containing methotrexate, doxorubicin, and cisplatin (MAP), 13.3% of the patients received additional ifosfamide and etoposide (MAPie), and 10.6% received additional interferon-alfa (MAPinf). Eighty-nine patients (78.8%) received all 12 courses according to protocol. Reasons for cancelled courses included ineffective treatment, poor physical conditions of the patient (whether or not caused by treatment toxicities), a patient’s own request, or death.

**Table 1:** Characteristics of 113 osteosarcoma patients.

Age at diagnosis in years, median (range)	15.0 (5.6–43.0)
Sex, number of males (%)	64 (56.6)
Self-reported ethnicity, number of Caucasians (%)	113 (100)
Metastases present at diagnosis, number of patients (%)	20 (17.7)
Treatment protocol, number of patients in EURAMOS-1 (%)	113 (100)
EURAMOS-1 treatment arm, number of patients (%)	
MAP	50 (44.2)
MAPie	15 (13.3)
MAPifn	12 (10.6)
Personalized	4 (3.5)
Unknown	32 (28.3)
MTX cumulative dose (12 g/m^2^ per course), number of patients	
12–72 g/m²	8
84–132 g/m²	16
144 g/m²	89

MAP, regimen of methotrexate, doxorubicin, cisplatin; MAPie, regimen of methotrexate, doxorubicin, cisplatin, ifosfamide, etoposide; MAPinf, regimen of methotrexate, doxorubicin, cisplatin, interferon-alfa.

### Genotyping

Copy number variants (n = 5), tri-allelic variants (n = 1), and X-chromosomal variants (n = 46) were not included in the analysis. QC resulted in exclusion of 90 variants due to unreliable cluster plots, 26 variants because of low call rate, 1,222 variants due to MAF<0.05, and one variant which deviated from HWE. One patient was excluded because of low overall sample call rate. A total of 113 patients and 545 variants were included for genetic association analyses (see **Supplementary Table S1** for the included variants). The Bonferroni corrected p-value threshold was <0.05/545 = 9.2 × 10^-5^.

### MTX Plasma Levels

Median MTX plasma level 48 h after MTX infusion was 0.25 µmol/L, ranging from 0.05 to 82.34 µmol/L (1,238 datapoints in 113 osteosarcoma patients) (**Table 2**). Weak but statistically significant correlations were found for MTX plasma levels with creatinine levels at t = 48 h (*r_s_*= 0.187, p = 1.8 × 10^-5^) and t = week 1 (*r_s_*= 0.163, p = 1.8 × 10^-6^), hemoglobin levels (*r_s_*= -0.190, p = 0.001) and with ALAT levels (*r_s_*= 0.102, p = 0.03) (**Table 3**). Neither sex, age, or cumulative MTX dose were associated with MTX plasma levels (data not shown) and were not included as covariate in the genetic association analyses. Thirty-three genetic variants in 20 genes were associated with MTX plasma levels at p<0.05 (**Supplementary Table S2**). Variant rs3736599 in *SULT1E1* passed the Bonferroni corrected *p*-value threshold [G vs. A allele; coef -0.31, (95% CI -0.46 - -0.17), p = 2.60×10^-5^] (**Table 5**).

**Table 2:** Characteristics of laboratory markers of nephrotoxicity, liver damage, and bone marrow toxicity in included osteosarcoma patients.

Measurement (in plasma)	Mean (SD) or median (range)*	Number of datapoints (of number of patients)
MTX level (t = 48 h), µmol/L	0.25 (0.05–82.34)	1238 (113)
Creatinine levels (t = 48 h), µmol/L	48.2 (15.1)	551 (91)
Creatinine levels (t = week 1), µmol/L	51.7 (17.9)	857 (110)
ALAT levels (t = 48 h), U/L	260 (10–4,677)	475 (87)
ASAT levels (t = 48 h), U/L	106 (11–4,420)	461 (87)
Hemoglobin levels (t = week 1), mmol/L	6.5 (0.8)	908 (112)
Thrombocyte counts (t = week 1), x 10^3^/mm³	184 (10–740)	896 (112)
Leukocyte counts (t = week 1), x 10^9^/L	4.4 (0.8–38.2)	905 (112)
Neutrophil counts (t = week 1), x 10^9^/L	2.3 (0.0–35.8)	709 (109)

*Mean and standard deviation (SD) for normally distributed data, median and range for other.

**Table 3:** Correlation of MTX plasma levels at 48 hours with toxicity markers.

Measurement (in plasma)	Spearman’s correlation coefficient	P-value
Creatinine level (t = 48 h)	**0.187**	**0.000018**
Creatinine level (t = week 1)	**0.163**	**0.0000018**
ALAT levels (t = 48 h)	0.102	**0.03**
ASAT levels (t = 48 h)	0.085	0.07
Hemoglobin levels (t = week 1)	**-0.109**	**0.001**
Thrombocyte counts (t = week 1)	-0.053	0.12
Leukocyte counts (t = week 1)	0.023	0.50
Neutrophil counts (t = week 1)	-0.033	0.38

Values depicted in bold are statistically significant (P-value < 0.05).

### Toxicity Markers

Availability and characteristics of collected toxicity markers are shown in **Table 2** (continuous outcomes) and **Table 4** (binary outcomes). Sex, age, and cumulative MTX dose were tested for association with each toxicity marker, and included as a covariate in multivariate genetic association analyses for that respective toxicity marker if associated (p < 0.05). A description of included covariates in the analysis of each toxicity marker, and an overview of results of genetic association analyses of both continuous and binary outcomes are provided in **Supplementary Tables S3–S10**.

**Table 4:** Characteristics of laboratory markers for liver damage and bone marrow toxicity, graded according to National Cancer Institute Common Terminology Criteria for Adverse Events (CTCAE) Version 5.0.

CTCAE term	Case-control designation	Cases, n of measurements (%)	Controls, n of measurements (%)
Alanine aminotransferase increased	CTCAE grade 0–1 vs. grade 2–4	358 (75.4)	177 (24.6)
Aspartate aminotransferase increased	CTCAE grade 0–1 vs. grade 2–4	237 (51.4)	224 (48.6)
Anemia	CTCAE grade 0–1 vs. grade 2–5	293 (32.3)	615 (67.7)
Platelet count decreased	CTCAE grade 0 vs. grade 1–4	351 (39.2)	545 (60.8)
White blood cell decreased	CTCAE grade 0–1 vs. grade 2–4	194 (21.4)	711 (78.6)
Neutrophil count decreased	CTCAE grade 0–2 vs. grade 3–4	87 (12.3)	622 (87.7)

The results of the genetic association analyses with continuous outcomes showed no genetic variants to be statistically significantly associated (p < 9.2 × 10^-5^) with creatinine levels, ASAT and ALAT, hemoglobin levels, leukocyte counts and neutrophil counts (**Supplementary Table S3–S10**). Association analysis of thrombocyte count (as a continuous variable) showed three statistically significantly associated variants in two genes (**Table 5**). These were rs4808326 in *CYP4F8* [G vs. A allele; coef 0.20, (95% CI 0.11-0.29), p = 2.91 × 10^-5^], rs4803418 in *CYP2B6* [C vs. G allele; coef -0.19, (95% CI -0.27 - -0.10), p = 3.04 × 10^-5^] and rs4803419 in *CYP2B6* [C vs. T allele, coef -0.19, (95% CI -0.28 - -0.09), p = 8.80 × 10^-5^].

For the nominally associated genetic variants (p < 0.05), association analyses using toxicity markers as a binary outcome (using the CTCAE graded toxicity measures) were performed to assess clinical relevance of these findings. This additional analysis was not possible for renal damage, as the vast majority of creatinine measurements were graded as grade 0. The analysis of the other binary outcomes did not yield additional variants surpassing the Bonferroni corrected p-value threshold. The statistically significant associations identified in the analysis with continuous data, being the association between thrombocyte counts and variants in *CYP2B6* and *CYP4F8*, did show a nominal significant association in binary analysis (p < 0.05) for two of the three identified variants, as depicted in **Table 5** (rs4808326 in *CYP4F8* and rs4803418 in *CYP2B6*).

**Table 5:** Genetic variants with a statistically significantly associated result (p < 9.2 × 10^-5^) in analysis with continuous outcomes, and corresponding results from analyses with binary outcomes.

Outcome	Gene	Variant	Chr	Position	Minor allele	MAF	Linear multivariate GEE analyses with continuous outcome				Logistic multivariate GEE analyses with binary outcome (CTCAE)			
							Coef	95% CI		P	Coef	95% CI		P
MTX plasma levels	*SULT1E1*	rs3736599	4	70,725,821	A	0.08	-0.31	-0.46	-0.17	0.00003	n.a.	n.a.	n.a.	n.a.
Thrombocyte counts	*CYP2B6*	rs4803418	19	41,511,803	G	0.31	-0.19	-0.27	-0.10	0.00003	0.32	0.01	0.63	0.046
Thrombocyte counts	*CYP4F8*	rs4808326	19	15,726,147	A	0.10	0.19	0.10	0.29	0.00006	-0.97	-1.55	-0.39	0.001
Thrombocyte counts	*CYP2B6*	rs4803419	19	41,512,792	T	0.32	-0.19	-0.28	-0.09	0.00009	0.32	0.00	0.65	0.051

MAF, minor allele frequency; GEE, generalized estimated equations; CTCAE, common terminology criteria of adverse events; MTX, methotrexate; Coef, coefficient; CI, confidence interval; P, p-value; n.a., not applicable.

## Discussion

This study identified statistically significant associations between four genetic variants in three drug metabolism genes and pharmacokinetic and toxicity markers derived from routine plasma measurements after HD-MTX infusion in osteosarcoma patients. These novel findings may add to further understanding of variation in the development of HD-MTX induced toxicities in osteosarcoma patients.

A variant in the 5’ untranslated region of the *sulfotransferase family 1E member 1* gene (*SULT1E1*, rs3736599) was statistically significantly associated with MTX plasma levels. In this cohort, carriers of the A allele had significantly lower MTX plasma levels at 48 h compared to carriers of the G allele. *SULT1E1* encodes the enzyme estrogen sulfotransferase, which is mainly responsible for sulfate conjugation of hormones and is known to be involved in metabolism of hormonal therapies (Whirl-Carrillo et al., 2012). *SULT1E1*, or other members of the sulfotransferase family, were not linked to MTX metabolism before, as MTX is not known to undergo sulfate conjugation. Variants in this gene family were also not found to be associated with MTX clearance in the GWAS in 434 patients with ALL by Trevino et al. (2009). Studies in rats have shown a potential link between MTX and sulfotransferases, as this drug was found to be a xenobiotic inducer of sulfotransferases expressed in the intestine and liver (Maiti and Chen, 2003; Chen et al., 2006). This could implicate a role for sulfotransferases in MTX elimination *via* the liver and intestines, tissues in which *SULT1E1* is also mainly expressed (Consortium, 2013). However, the identified variant in this study (rs3736599) is, due to its location in the 5’UTR region, not known to have a direct impact on SULT1E1 protein function, or to be an expression quantitative trait locus (eQTL), meaning it is not associated to the expression level of *SULT1E1*, or any other gene in any tissue (Consortium, 2013).

Three variants in two genes were statistically significantly associated with thrombocyte counts after HD-MTX using continuous outcome measures in a linear regression model, being two variants (rs4803418 and rs4803419) in *cytochrome P450 family 2 subfamily B member 6* (*CYP2B6)* and one variant (rs4808326) in *cytochrome P450 family 4 subfamily F member 8* (*CYP4F8*). Secondary analysis of these variants using a binary approach with CTCAE graded toxicity data resulted in a nominal significant associations (p < 0.05). Although not surpassing the Bonferroni-corrected p-value threshold (most likely due to decreased power), this result does underline the potential clinical relevance of these findings. This also indicates that the primary analyses, with an approach using continues outcomes, indeed aids in the identification of genetic variants that might have clinical relevance.

The variants in *CYP2B6* (rs4803418 and rs4803419) are located in an intronic region, and are in high linkage disequilibrium (r^2^ = 0.88), therefore likely represent the same locus (Machiela and Chanock, 2015). Carriers of the variant alleles (G allele in rs4803418 and T allele in rs4803419) showed lower thrombocyte counts after HD-MTX courses compared to patients carrying the reference alleles. *CYP2B6* is involved in the metabolism of many drugs, e.g. efavirenz, cyclophosphamide, and bupropion (Zanger and Klein, 2013). Both identified (intronic) variants are not known to have an direct effect on protein function, but are significant eQTLs for expression of *CYP2B6* in the transverse colon (Consortium, 2013). Interestingly, evidence exists that a missense variant in *CYP2B6* (rs3211371) has an influence on efficacy of the thrombocyte aggregation inhibitor clopidogrel (Kassimis et al., 2012). The underlying mechanism for this association was hypothesized to be a pharmacokinetic effect on clopidogrel metabolism. This association was not replicated by others, nor were associations with other variants in *CYP2B6* identified (Cresci et al., 2014; Gonzalez et al., 2016). Both our identified variants were not investigated in relation to clopidogrel or other phenotypes related to thrombocytes. The variants in *CYP2B6* were also nominally associated (p < 0.05) with leucocytes and neutrophils (**Supplementary Tables S9 and S10**, respectively). As variants in *CYP2B6* were not associated to MTX pharmacokinetics in our study or previous studies, one could hypothesize there is a potential effect of the CYP2B6 protein on myelopoiesis. However, *CYP2B6* is expressed mainly in the liver, but not in the bone marrow or whole blood, leaving the underlying mechanism of these associations unclear (Consortium, 2013).

The third statistically significant associated variant in our toxicity analyses was the intronic variant rs4808326 in *CYP4F8*. Carriers of the A allele had higher thrombocyte counts after MTX infusion compared to those carrying the G allele. *CYP4F8* codes for an enzyme known to be involved in metabolism of prostaglandins, leukotrienes and long chain fatty acids, and is mainly expressed in prostate and skin (Kirischian and Wilson, 2012). This gene is not known to have a prominent role in drug metabolism and was not linked to MTX or thrombocyte counts before. rs4808326 is a significant eQTL for expression of other CYP4F subfamily members, including *CYP4F12, CYP4F24P*, and *CYP4F3*, in multiple tissues (Consortium, 2013). However, to our knowledge, none of the CYP4F subfamily members were previously implicated in MTX pharmacokinetics or MTX-induced toxicities, nor were these eQTLs present in liver, kidneys or bone marrow. A GWAS on mean platelet volume and thrombocyte count in 66,867 healthy Caucasian individuals by Gieger *et al.* showed no genome-wide significant association with genetic variants in *CYP4F8* (or our other top hits in *CYP2B6*), suggesting that our identified associations were MTX-induced effects (Gieger et al., 2011).

To get more insight in the effect of the identified variants on gene function we used RegulomeDB, which annotates variants with known and predicted regulatory elements (Boyle et al., 2012). Here, multiple public data sources are used to generate a predictive score, where a higher score indicates that a variant is more likely to have regulatory impact. According to this database, the two identified variants in *CYP2B6* are not likely to have a regulatory effect (RegulomeDB score of 0.18 for both variants), whereas the variants in *CYP4F8* and *SULT1E1* have higher score (RegulomeDB scores of 0.52 and 0.61, respectively), suggesting a potential regulatory effect more specifically as indicated by the database *via* transcription binding sides and DNase peaks.

Genetic variation associated with HD-MTX efficacy or toxicity has been studied in the past. To date, pharmacogenetic studies in this field have resulted in genetic variants annotated with level 2A evidence at highest, meaning there is moderate evidence for the variant-drug combination but no clinical implementation yet (Whirl-Carrillo et al., 2012). These level 2A variants include rs1045642 in *ABCB1*, rs11045879 in *SLCO1B1*, rs1801133 in *MTHFR*, and rs1801394 in *MTRR* and rs4673993 in *ATIC* with MTX pharmacokinetics or toxicity outcomes. *MTHFR*, *MTRR*, and *ATIC* were not present on the DMET array used in our study. The previously identified variant rs1045642 in *ABCB1* was included in our genetic association analyses, showing no association (p = 0.430) with MTX plasma levels, nor with any of the toxicity markers. Multiple candidate gene studies found this variant to be associated to MTX plasma levels and different MTX-induced toxicities in patients with hematological malignancies (Suthandiram et al., 2014; Zgheib et al., 2014; Gregers et al., 2015; Tsujimoto et al., 2016; Liu et al., 2017). Potential contributors to contrasting findings are differences in outcomes, different populations with different genetic backgrounds, and different HD-MTX treatment regimens (2 – 5 g/m² in hematological malignancies compared to 12 g/m² in osteosarcoma). A prospective candidate gene study investigating the relation of genetic variants and HD-MTX pharmacokinetics in osteosarcoma patients identified statistically significant associations between MTX clearance and variants in *ABCG2* and *UGT1A* (Lui et al., 2018). *ABCG2* was not included in this study but the identified variant in *UGT1A* (rs4148324) was in high LD (r² > 0.90) with three variants in the present study (rs887829, rs111741722, rs10929302), but these variants showed no association with MTX 48 h plasma levels (using both an additive and dominant model, data not shown). The discrepancy in results is most likely due to major differences in study design and outcome measures.

The only GWAS on MTX-related phenotypes to date identified an association between variant rs11045879 in *SLCO1B1* and MTX clearance in patients with ALL, which was replicated by others (Trevino et al., 2009; Lopez-Lopez et al., 2011; Radtke et al., 2013; Ramsey et al., 2013; Csordas et al., 2014; Goricar et al., 2014; Zhang et al., 2014; Liu et al., 2017). *SLCO1B1* codes for a transporter known for its function of transporting MTX into the hepatocyte (Mikkelsen et al., 2011). Unfortunately, rs11045879 was not present on the DMET array, so its association with MTX pharmacokinetic and toxicity markers was not directly investigated in our cohort. However, 18 other variants in *SLCO1B1* were present, including variant rs4149056 which is in high LD with rs11045879 (r^2^ = 0.92) (Machiela and Chanock, 2015). We did not identify an association with this variant and MTX 48 h plasma levels (p = 0.211). A possible explanation might be a difference in outcome measure, as the original GWAS used multiple MTX plasma levels to calculate average MTX clearance, compared to MTX 48 h plasma levels in this study. Additionally, the limited sample size could have resulted in insufficient power to detect this association with a relatively low allele frequency in this cohort (MAF = 0.16). Despite the result not being significant, the direction of association is consistent with previous findings, being higher MTX 48 h plasma levels in case of carrying the (C) allele (coef 0.112) (Trevino et al., 2009; Lopez-Lopez et al., 2011; Radtke et al., 2013; Ramsey et al., 2013; Csordas et al., 2014; Goricar et al., 2014; Zhang et al., 2014; Liu et al., 2017). Our analyses did show an association with levels of liver enzyme ALAT after HD-MTX infusion (p = 0.007, uncorrected for multiple testing) with the variants rs4149056 and rs11045819 in *SLCO1B1*. As this gene is a highly expressed transporter on the basolateral membrane of hepatocytes with MTX as its substrate, one could hypothesize that its activity has an influence on the intracellular accumulation of MTX in hepatocytes, and therefore could facilitate liver damage (Mikkelsen et al., 2011).

MTX induced toxicities typically develop in a dose-dependent fashion. Therefore, correlations were calculated between MTX plasma levels and the different toxicity markers in this study. The observed directions of effects were consistent with expectations, except for the positive correlation with leukocytes. Hemoglobin, thrombocyte, and neutrophil counts showed a negative correlation with MTX levels, indicating more bone marrow suppression with higher MTX plasma levels. Creatinine, ALAT, and ASAT showed a positive correlation with MTX plasma levels, indicating more nephrotoxicity and hepatotoxicity with higher MTX plasma levels. Some correlations were statistically significant, but all correlations were too weak for clinical relevance. The strongest significant correlation was observed for MTX plasma levels and creatinine levels, 48 h after MTX infusion. This corresponds with the results of Tsuruwasa *et al.*, as they found significant associations of MTX plasma levels with nephrotoxicity, but not with hepatotoxicity or infections (Tsurusawa et al., 2015). Taking into account more extensive information, for example measuring metabolite levels (e.g. 7-OH-MTX), might result in more accurate predictions of nephrotoxicity or hepatic toxicity (Holmboe et al., 2012; Csordas et al., 2013).

The present study aimed to gain insight in the relevance of ADME gene variation for MTX metabolism, but had its limitations. Firstly, the power of this study was limited due to a modest number of patients and independent data points. Secondly, this study focused on phenotypes that are routinely measured in clinical practice to obtain reliable repeated measures after each MTX course. These continuous measures give insight in the MTX-induced metabolism changes in a patient, but are no direct measures of toxicities. Some other clinically relevant MTX-induced adverse events, including e.g. mucositis and encephalopathy, were not included in the present study. Reason for this is that these outcomes could not be retrospectively collected in a reliable way. However, regarding the clinical relevance of these adverse events, future prospective studies to investigate these adverse events in more detail will be certainly of value. Finally, osteosarcoma patients receive a multidrug chemotherapeutic regimen, and although the other chemotherapeutic drugs are not given simultaneously with MTX, they could have an impact on laboratory results measured, especially in later stages of the treatment. Drugs with known interactions with MTX, e.g. trimethoprim-sulfamethoxazole, were not administered close to or together with MTX according to protocol. However, other drugs with unknown interactions may have influenced MTX clearance *via* impaired renal function or may directly influence the laboratory results that were used as outcome measures. These interactions were not mentioned in the protocols and as a consequence, these were not taken into account in this study.

In conclusion, this study identified statistically significant associations for a genetic variant in *SULT1E1* with MTX plasma levels and three genetic variants in *CYP2B6* and *CYP4F8* with thrombocyte counts after HD-MTX infusion in osteosarcoma patients. These variants were not previously described to play a role in MTX pharmacokinetics. Replication of these findings and pinpointing the exact role of these variants and genes in MTX metabolism and development of toxicities will improve understanding the interpatient variability in MTX response and may ultimately provide new opportunities to optimize treatment for osteosarcoma patients.

## Data Availability Statement

The raw data supporting the conclusions of this article will be made available by the authors, without undue reservation.

## Ethics Statement

The studies involving human participants were reviewed and approved by the institutional review board of the Radboud University Medical Center (Commissie Mensgebonden Onderzoek Regio Arnhem Nijmegen), and the institutional ethics committees for inclusion of patients in Groningen, Leiden and Amsterdam. Written informed consent to participate in this study was provided by the participants’ legal guardian/next of kin.

## Author Contributions

EH, MK, SV, MH, H-JG, MC, and ML wrote the manuscript. MC and ML designed the research. ML, MH, UF, BS, HG, H-JG, and JB provided the patient material. MK collected the clinical data. EH, MK, and SV performed the statistical analyses. All authors contributed to the article and approved the submitted version.

## Funding

This work was supported by the Princess Máxima Center Foundation.

## Conflict of Interest

The authors declare that the research was conducted in the absence of any commercial or financial relationships that could be construed as a potential conflict of interest.
