# Supplementary material for: Analysis of Drug Metabolizing Gene Panel in Osteosarcoma Patients Identifies Association Between Variants in SULT1E1, CYP2B6 and CYP4F8 and Methotrexate Levels and Toxicities
Source: Front Pharmacol. 2020 Aug 12;11:1241. doi: 10.3389/fphar.2020.01241 (PMC7435008; doi:10.3389/fphar.2020.01241)
Supplement: Supplementary file 1 [file DataSheet_1.pdf]

Table S1. 545 genetic variants in 176 genes included in genetic association analyses

Gene	SNP	Gene	SNP	Gene	SNP	Gene	SNP	Gene	SNP	Gene	SNP
ABCB1	rs10276036	*ADH7*	rs1573496	*CYP19A1*	rs700518	*EPHX1*	rs1051740	*PPARD*	rs1003973	*SLC7A7*	rs1805059
ABCB1	rs1045642	*ADH7*	rs971074	*CYP1A2*	rs2470890	*EPHX1*	rs1051741	*PPARD*	rs1883322	*SLC7A7*	rs1805061
ABCB1	rs1128503	*AHR*	rs10249788	*CYP1A2*	rs35694136	*EPHX1*	rs2292566	*PPARD*	rs2038067	*SLC7A7*	rs2281677
ABCB1	rs17064	*AHR*	rs2066853	*CYP1A2*	rs762551	*EPHX2*	rs41507953	*PPARD*	rs2076167	*SLC7A7*	rs8018462
ABCB1	rs2032588	*AKAP9*	rs2049900	*CYP1B1*	rs1056836	*FAAH*	rs324420	*PPARD*	rs2267665	*SLC7A8*	rs1884545
ABCB1	rs2214102	*AKAP9*	rs7785971	*CYP1B1*	rs1056837	*FMO1*	rs1126692	*PPARD*	rs2267667	*SLC7A8*	rs2236135
ABCB1	rs2235013	*ALB*	rs3756067	*CYP1B1*	rs1800440	*FMO1*	rs12954	*PPARD*	rs2267668	*SLC7A8*	rs2268873
ABCB1	rs2235033	*ALDH1A1*	rs13959	*CYP20A1*	rs1048013	*FMO1*	rs742350	*PPARD*	rs2267669	*SLC7A8*	rs2268877
ABCB1	rs2235040	*ALDH2*	rs886205	*CYP24A1*	rs2296241	*FMO1*	rs7877	*PPARD*	rs6457816	*SLC7A8*	rs7141505
ABCB1	rs3842	*ALDH3A1*	rs887241	*CYP24A1*	rs2762934	*FMO2*	rs2020860	*PPARD*	rs6901410	*SLC7A8*	rs8013529
ABCB1	rs9282564	*AOX1*	rs11678615	*CYP24A1*	rs6068816	*FMO2*	rs2020861	*PPARD*	rs6906237	*SLC7A8*	rs910795
ABCB11	rs2287622	*AOX1*	rs11684227	*CYP26A1*	rs10882140	*FMO2*	rs2020863	*PPARD*	rs6922548	*SLCO1A2*	rs10841795
ABCB11	rs3755163	*AOX1*	rs16834034	*CYP26A1*	rs4418728	*FMO2*	rs2020865	*PPARD*	rs7739752	*SLCO1A2*	rs4078
ABCB11	rs3770602	*AOX1*	rs3731722	*CYP26A1*	rs7905939	*FMO2*	rs2020868	*PPARD*	rs7751481	*SLCO1A2*	rs7957203
ABCB11	rs3770603	*AOX1*	rs6729738	*CYP2A13*	rs1709082	*FMO2*	rs2020869	*PPARG*	rs1152003	*SLCO1B1*	rs11045819
ABCB11	rs4148768	*AOX1*	rs7563682	*CYP2A6*	rs1137115	*FMO2*	rs2020870	*PPARG*	rs1801282	*SLCO1B1*	rs2291075
ABCB11	rs4148771	*APOA2*	rs5085	*CYP2A6*	rs28399433	*FMO2*	rs2307492	*PPARG*	rs3856806	*SLCO1B1*	rs2306283
ABCB11	rs4148771	*ARNT*	rs2228099	*CYP2A6*	rs4803381	*FMO2*	rs28369860	*PPARG*	rs9833097	*SLCO1B1*	rs4149056
ABCB11	rs4148776	*ARSA*	rs131713	*CYP2A6*	rs8192729	*FMO2*	rs7512785	*PTGIS*	rs5629	*SLCO1B1*	rs4149057
ABCB11	rs4668115	*ARSA*	rs743616	*CYP2A7*	rs3869579	*FMO2*	rs7515157	*QPRT*	rs13331798	*SLCO1B3*	rs2053098
ABCB11	rs473351	*ATP7B*	rs1051332	*CYP2B6*	rs2279343	*FMO3*	rs1736557	*QPRT*	rs3862476	*SLCO1B3*	rs3764006
ABCB11	rs495714	*ATP7B*	rs1061472	*CYP2B6*	rs3745274	*FMO3*	rs1800822	*RALBP1*	rs10898	*SLCO1B3*	rs4149117
ABCB11	rs496550	*ATP7B*	rs1801243	*CYP2B6*	rs4803418	*FMO3*	rs2066534	*RALBP1*	rs12680	*SLCO1B3*	rs7311358
ABCB11	rs497692	*ATP7B*	rs1801244	*CYP2B6*	rs4803419	*FMO3*	rs2266780	*RALBP1*	rs3322	*SLCO3A1*	rs1517618
ABCB11	rs7602171	*ATP7B*	rs1801249	*CYP2B6*	rs8192719	*FMO3*	rs2266782	*RPL13*	rs9930567	*SLCO3A1*	rs2190748
ABCB4	rs1202283	*ATP7B*	rs2277448	*CYP2C18*	rs2281891	*FMO3*	rs909530	*SLC10A1*	rs4646285	*SLCO3A1*	rs2283458
ABCB4	rs2097937	*ATP7B*	rs732774	*CYP2C18*	rs2860840	*FMO4*	rs2223477	*SLC10A2*	rs188096	*SLCO3A1*	rs3743369
ABCB4	rs2109505	*CA5P*	rs11150564	*CYP2C19*	rs12248560	*FMO5*	rs894469	*SLC10A2*	rs2301157	*SLCO3A1*	rs960440
ABCB4	rs2230028	*CA5P*	rs11859842	*CYP2C19*	rs4244285	*FMO6*	rs1736565	*SLC10A2*	rs2301159	*SLCO4A1*	rs2236553

ABCB4	rs2302387	*CA5P*	rs1364182	*CYP2C8*	rs10509681	*FMO6*	rs7886938	*SLC10A2*	rs279941	*SLCO4A1*	rs3787537
ABCB4	rs3747806	*CBR1*	rs1005695	*CYP2C8*	rs1058930	*FMO6*	rs7889839	*SLC10A2*	rs279942	*SLCO5A1*	rs1138541
ABCB4	rs4148805	*CBR1*	rs2835265	*CYP2C8*	rs11572080	*GSTA1*	rs4715332	*SLC10A2*	rs3803258	*SLCO5A1*	rs16936279
ABCB4	rs4148808	*CBR1*	rs3787728	*CYP2D6*	rs1058164	*GSTA2*	rs1803684	*SLC10A2*	rs7987433	*SLCO5A1*	rs3750266
ABCC1	rs212090	*CBR1*	rs998383	*CYP2D6*	rs1065852	*GSTA2*	rs2180314	*SLC13A1*	rs1880179	*SPG7*	rs12960
ABCC1	rs212091	*CBR3*	rs1056892	*CYP2D6*	rs1080983	*GSTA2*	rs6577	*SLC13A1*	rs2140516	*SPG7*	rs2292954
ABCC1	rs246221	*CBR3*	rs2835286	*CYP2D6*	rs1080985	*GSTA3*	rs512795	*SLC13A1*	rs2204295	*SPN*	rs4788172
ABCC1	rs4148380	*CBR3*	rs8133052	*CYP2D6*	rs1135840	*GSTA4*	rs13197674	*SLC13A1*	rs6962039	*SULT1B1*	rs11249460
ABCC1	rs8187858	*CCDC101*	rs11401	*CYP2D6*	rs16947	*GSTA4*	rs1802061	*SLC15A1*	rs1339067	*SULT1B1*	rs11731028
ABCC2	rs1137968	*CDA*	rs1048977	*CYP2D6*	rs28360521	*GSTA4*	rs367836	*SLC15A1*	rs2297322	*SULT1B1*	rs1604741
ABCC2	rs17222723	*CDA*	rs2072671	*CYP2D6*	rs3892097	*GSTA4*	rs405729	*SLC15A2*	rs1143670	*SULT1C4*	rs1402467
ABCC2	rs2273697	*CDA*	rs818202	*CYP2E1*	rs2070673	*GSTA4*	rs7496	*SLC15A2*	rs1143671	*SULT1E1*	rs1881668
ABCC2	rs3740066	*CES2*	rs11568311	*CYP2E1*	rs2515641	*GSTA5*	rs4715354	*SLC15A2*	rs1143672	*SULT1E1*	rs3736599
ABCC2	rs717620	*CES2*	rs11568314	*CYP2F1*	rs305968	*GSTA5*	rs7748890	*SLC15A2*	rs2257212	*SULT1E1*	rs3775770
ABCC2	rs8187707	*CES2*	rs4783745	*CYP2F1*	rs58285195	*GSTM2*	rs530021	*SLC15A2*	rs2293616	*SULT1E1*	rs3822172
ABCC3	rs1051640	*CHST1*	rs2028985	*CYP2J2*	rs890293	*GSTM2*	rs592792	*SLC16A1*	rs1049434	*SULT2B1*	rs1132054
ABCC3	rs11568591	*CHST1*	rs750398	*CYP2S1*	rs338599	*GSTM3*	rs1799735	*SLC16A1*	rs12727968	*SULT2B1*	rs2302948
ABCC3	rs2277624	*CHST1*	rs895729	*CYP39A1*	rs2277119	*GSTM3*	rs2234696	*SLC16A1*	rs7169	*SULT2B1*	rs2544794
ABCC3	rs4148416	*CHST1*	rs9787901	*CYP39A1*	rs7761731	*GSTM3*	rs7483	*SLC16A1*	rs9429505	*SULT4A1*	rs138056
ABCC4	rs1059751	*CHST10*	rs1530031	*CYP3A4*	rs2242480	*GSTM5*	rs11807	*SLC22A1*	rs1867351	*SULT4A1*	rs138057
ABCC4	rs1189466	*CHST10*	rs3748930	*CYP3A43*	rs17342647	*GSTM5*	rs1296954	*SLC22A1*	rs628031	*TPSG1*	rs909921
ABCC4	rs1678339	*CHST10*	rs3828193	*CYP3A43*	rs533486	*GSTO1*	rs4925	*SLC22A1*	rs683369	*UGT1A1*	rs1042640
ABCC4	rs2274405	*CHST11*	rs2463018	*CYP3A43*	rs800667	*GSTP1*	rs1138272	*SLC22A1*	rs72552763	*UGT1A1*	rs10929302
ABCC4	rs2274406	*CHST11*	rs2463437	*CYP3A5*	rs776746	*GSTP1*	rs1695	*SLC22A11*	rs1783811	*UGT1A1*	rs10929303
ABCC4	rs2274407	*CHST11*	rs2468110	*CYP3A7*	rs2257401	*GSTZ1*	rs1046428	*SLC22A11*	rs2078267	*UGT1A1*	rs111741722
ABCC4	rs3742106	*CHST11*	rs7847	*CYP4A11*	rs11211402	*GSTZ1*	rs7972	*SLC22A12*	rs11231825	*UGT1A1*	rs3755319
ABCC4	rs4148551	*CHST11*	rs903247	*CYP4B1*	rs2297809	*GSTZ1*	rs7975	*SLC22A13*	rs4679028	*UGT1A1*	rs4124874
ABCC4	rs4148553	*CHST13*	rs1056522	*CYP4B1*	rs2297810	*HMGCR*	rs5909	*SLC22A13*	rs9842091	*UGT1A1*	rs8330
ABCC5	rs3749442	*CHST13*	rs1873397	*CYP4B1*	rs3215983	*HNMT*	rs1050891	*SLC22A14*	rs149738	*UGT1A1*	rs887829
ABCC5	rs3805114	*CHST13*	rs3856650	*CYP4B1*	rs4646487	*HNMT*	rs4245861	*SLC22A14*	rs171248	*UGT1A3*	rs6706232
ABCC5	rs562	*CHST13*	rs6783962	*CYP4B1*	rs4646491	*HNMT*	rs4646333	*SLC22A14*	rs183574	*UGT1A4*	rs6755571
ABCC5	rs7636910	*CHST2*	rs3755739	*CYP4F11*	rs1060463	*MAT1A*	rs17102596	*SLC22A2*	rs316003	*UGT1A6*	rs1105880

ABCC5	rs939336	*CHST2*	rs4683739	*CYP4F11*	rs2305801	*MAT1A*	rs4934027	*SLC22A2*	rs316019	*UGT1A7*	rs7586110
ABCC6	rs2238472	*CHST2*	rs6664	*CYP4F11*	rs3765070	*MAT1A*	rs7087728	*SLC22A2*	rs624249	*UGT1A9*	rs3832043
ABCC6	rs8058694	*CHST3*	rs1871450	*CYP4F11*	rs8104361	*MAT1A*	rs9285726	*SLC22A3*	rs2292334	*UGT2A1*	rs11249454
ABCC6	rs8058696	*CHST3*	rs4148943	*CYP4F12*	rs593421	*NAT1*	rs4986993	*SLC22A3*	rs668871	*UGT2A1*	rs2288741
ABCC8	rs757110	*CHST3*	rs4148944	*CYP4F12*	rs609290	*NAT2*	rs1041983	*SLC22A4*	rs1050152	*UGT2A1*	rs4148301
ABCG1	rs1044317	*CHST3*	rs4148945	*CYP4F12*	rs609636	*NAT2*	rs1208	*SLC22A4*	rs272893	*UGT2A1*	rs4148304
ABCG1	rs1541290	*CHST3*	rs4148946	*CYP4F12*	rs688755	*NAT2*	rs1799929	*SLC22A5*	rs1045020	*UGT2B15*	rs1902023
ABCG1	rs3788007	*CHST3*	rs4148949	*CYP4F2*	rs2074900	*NAT2*	rs1799930	*SLC22A5*	rs274548	*UGT2B15*	rs3100
ABCG1	rs3788010	*CHST3*	rs4148950	*CYP4F2*	rs2108622	*NAT2*	rs1799931	*SLC22A5*	rs274558	*UGT2B15*	rs4148269
ABCG1	rs425215	*CHST3*	rs730720	*CYP4F2*	rs3093106	*NAT2*	rs1801280	*SLC22A7*	rs2242416	*UGT2B17*	rs28374627
ABCG1	rs492338	*CHST3*	rs731027	*CYP4F2*	rs3093153	*NNMT*	rs2852425	*SLC22A7*	rs2270860	*UGT2B4*	rs1131878
ABCG1	rs914189	*CHST5*	rs2641806	*CYP4F8*	rs2056822	*NQO1*	rs10517	*SLC22A8*	rs2276299	*UGT2B4*	rs13119049
ABCG2	rs2231142	*CHST5*	rs2738792	*CYP4F8*	rs4239614	*NQO1*	rs1800566	*SLC25A27*	rs9381468	*UGT2B4*	rs13142440
ABP1	rs1049793	*COMT*	rs4633	*CYP4F8*	rs4646523	*NQO1*	rs689453	*SLC25A27*	rs953062	*UGT2B4*	rs1966151
ABP1	rs10893	*COMT*	rs4680	*CYP4F8*	rs4808326	*NR1I2*	rs2276707	*SLC28A1*	rs2242046	*UGT2B7*	rs28365062
ABP1	rs12179	*CROT*	rs31651	*CYP4Z1*	rs7512729	*NR1I2*	rs3814055	*SLC28A1*	rs2242048	*UGT2B7*	rs7438284
ABP1	rs12539	*CROT*	rs31652	*CYP51A1*	rs7793861	*NR1I2*	rs6785049	*SLC28A1*	rs2290272	*UGT2B7*	rs7439366
ADH1A	rs12512110	*CROT*	rs7785206	*CYP51A1*	rs7797834	*NR1I3*	rs11584174	*SLC28A1*	rs2305367	*UGT2B7*	rs7662029
ADH1A	rs1826909	*CYP11B1*	rs1134095	*CYP7A1*	rs12542233	*NR1I3*	rs2501870	*SLC28A1*	rs8025045	*UGT2B7*	rs7668258
ADH1A	rs6811453	*CYP11B1*	rs4736312	*CYP7A1*	rs13251066	*NR1I3*	rs55802895	*SLC28A1*	rs8187758	*VKORC1*	rs17708472
ADH1C	rs1789915	*CYP11B1*	rs5297	*CYP7A1*	rs3808607	*NR3C1*	rs6196	*SLC28A2*	rs1060896	*VKORC1*	rs2359612
ADH4	rs1126670	*CYP11B1*	rs5303	*CYP7A1*	rs8192879	*PGAP3*	rs2952151	*SLC28A3*	rs10868138	*VKORC1*	rs2884737
ADH4	rs1126671	*CYP11B1*	rs7003319	*CYP7B1*	rs6980478	*PON1*	rs854560	*SLC28A3*	rs7853758	*VKORC1*	rs7294
ADH4	rs1126672	*CYP11B2*	rs4543	*CYP7B1*	rs6987861	*PON3*	rs11764079	*SLC28A3*	rs7867504	*VKORC1*	rs8050894
ADH4	rs3762894	*CYP17A1*	rs6162	*CYP8B1*	rs6771233	*PON3*	rs11770903	*SLC5A6*	rs1395	*VKORC1*	rs9923231
ADH5	rs1154400	*CYP17A1*	rs6163	*CYP8B1*	rs6774801	*PON3*	rs13226149	*SLC5A6*	rs7081	*VKORC1*	rs9934438
ADH5	rs2602836	*CYP17A1*	rs743572	*CYP8B1*	rs735320	*PON3*	rs17882539	*SLC6A6*	rs2341970	*XDH*	rs1884725
ADH6	rs10002894	*CYP19A1*	rs10046	*DPYD*	rs17376848	*PON3*	rs2072200	*SLC6A6*	rs9036	*XDH*	rs207440
ADH6	rs10008281	*CYP19A1*	rs1062033	*DPYD*	rs1801265	*POR*	rs17685	*SLC7A5*	rs1060253	*XDH*	rs2295475
ADH6	rs6830685	*CYP19A1*	rs4646	*DPYD*	rs2297595	*POR*	rs6965343	*SLC7A7*	rs1061040		

SNP, single nucleotide polymorphism

Table S2. Genetic variants associated with MTX plasma levels, 48 hours after MTX infusion (P<0.05)

Gene	SNP	Chr	Position	Minor allele	MAF	Coefficient	95% CI		P
SULT1E1	**rs3736599**	**4**	**70,725,821**	**A**	**0.08**	**-0.31**	**-0.46**	**-0.17**	**0.00003**
SLC6A6	rs2341970	3	14,474,225	T	0.25	-0.21	-0.33	-0.10	0.0004
CYP1B1	rs1056837	2	38,298,150	T	0.42	-0.17	-0.27	-0.07	0.0007
CYP1B1	rs1056836	2	38,298,203	G	0.42	-0.16	-0.26	-0.07	0.0008
GSTM2	rs530021	1	110,210,780	C	0.06	-0.27	-0.43	-0.10	0.002
SLC22A2	rs316003	6	160,645,832	G	0.19	-0.18	-0.30	-0.06	0.003
ABCB11	rs4148776	2	169,870,882	C	0.08	-0.21	-0.35	-0.06	0.006
CHST1	rs2028985	11	45,695,362	A	0.07	-0.25	-0.43	-0.07	0.007
CHST10	rs1530031	2	101,009,326	A	0.47	-0.16	-0.28	-0.04	0.007
CHST10	rs3828193	2	101,031,561	C	0.48	-0.13	-0.22	-0.04	0.007
CHST10	rs3748930	2	101,010,082	C	0.47	-0.16	-0.27	-0.04	0.008
PPARD	rs6901410	6	35,330,030	C	0.06	-0.24	-0.43	-0.05	0.013
GSTP1	rs1138272	11	67,353,579	T	0.11	-0.18	-0.33	-0.04	0.013
ABCB11	rs3755163	2	169,875,539	A	0.05	-0.21	-0.37	-0.04	0.017
ABCB11	rs4148771	2	169,875,534	delCT	0.05	-0.20	-0.37	-0.03	0.020
PPARD	rs6922548	6	35,353,523	G	0.06	-0.22	-0.40	-0.03	0.022
PPARD	rs7739752	6	35,339,035	T	0.06	-0.22	-0.40	-0.03	0.022
NR1I2	rs2276707	3	119,534,153	T	0.18	-0.14	-0.26	-0.02	0.022
CYP2C8	rs1058930	10	96,818,119	G	0.06	0.45	0.05	0.85	0.026
CYP2S1	rs338599	19	41,700,493	G	0.05	-0.22	-0.41	-0.02	0.027
SLC22A14	rs149738	3	38,367,984	G	0.46	0.11	0.01	0.22	0.031
SLC22A14	rs183574	3	38,363,837	C	0.46	0.11	0.01	0.22	0.031
SLC22A14	rs171248	3	38,363,393	C	0.46	0.11	0.01	0.22	0.031
GSTM3	rs1799735	1	110,280,254	delAGG	0.12	-0.17	-0.32	-0.01	0.035
POR	rs17685	7	75,616,105	A	0.29	-0.12	-0.23	0.00	0.041
SLC22A2	rs624249	6	160,679,400	T	0.42	0.14	0.01	0.28	0.042
ABCB4	rs2097937	7	87,030,903	C	0.24	-0.15	-0.30	-0.01	0.042
ABCB11	rs4148768	2	169,887,154	T	0.09	0.23	0.01	0.44	0.044
ABCC6	rs8058696	16	16,278,869	C	0.42	-0.10	-0.20	0.00	0.044
CYP2D6	rs1065852	22	42,526,694	T	0.23	-0.13	-0.25	0.00	0.046
CYP2A13	rs1709082	19	41,601,609	G	0.07	0.30	0.01	0.60	0.046
CYP2D6	rs28360521	22	42,528,976	A	0.24	-0.12	-0.23	0.00	0.048

MTX, methotrexate; SNP, single nucleotide polymorphism; Chr, chromosome; MAF, minor allele frequency; 95% CI, 95% confidence interval; P, p-value; del, deletion.

Covariate included in statistical model is creatinine plasma levels at week 1.

Effect sizes and 95% CI are reported for the minor allele.

A coefficient below 0 indicates that carriers of minor allele have lower MTX plasma levels compared to carriers of the major allele.

The variant depicted in bold surpasses the Bonferroni corrected p-value threshold of 9.2×10^{-5}.

The following variants are in linkage disequilibrium (r^2>0.80): rs3755163 and rs4148771 (*ABCB11*), all depicted variants within *CHST10*, *CYP1B1*, *SLC22A14*, *PPARD* and *CYP2D6*.

Table S3. Genetic variants associated with creatinine plasma levels, 48 hours after MTX infusion (P<0.05)

Gene	SNP	Chr	Position	Minor allele	MAF	Coefficient	95% CI		P
ABCC1	rs246221	16	16,138,322	C	0.25	-4.86	-7.82	-1.89	0.001
VKORC1	rs7294	16	31,102,321	A	0.33	-4.51	-7.30	-1.73	0.001
GSTA1	rs4715332	6	52,669,185	G	0.42	5.68	2.13	9.24	0.002
ABCB4	rs2230028	7	87,056,176	G	0.11	-5.40	-9.16	-1.65	0.005
CYP3A43	rs17342647	7	99,459,256	T	0.08	7.62	2.32	12.92	0.005
CROT	rs7785206	7	87,021,023	C	0.10	-5.89	-10.01	-1.78	0.005
SLCO3A1	rs3743369	15	92,707,569	G	0.42	-4.51	-7.69	-1.32	0.006
SLC22A7	rs2242416	6	43,273,604	A	0.43	4.90	1.37	8.44	0.007
GSTA5	rs4715354	6	52,708,797	T	0.47	-3.87	-6.70	-1.04	0.007
CDA	rs1048977	1	20,945,055	T	0.34	3.62	0.90	6.34	0.009
ADH1A	rs12512110	4	100,195,815	A	0.08	6.22	1.53	10.90	0.009
VKORC1	rs9923231	16	31,107,689	A	0.47	4.27	1.05	7.50	0.009
VKORC1	rs8050894	16	31,104,509	C	0.48	4.12	1.01	7.23	0.009
VKORC1	rs9934438	16	31,104,878	T	0.47	4.18	1.02	7.35	0.010
ABCB11	rs2287622	2	169,830,328	T	0.41	4.90	1.05	8.76	0.013
SLC15A1	rs2297322	13	99,376,181	A	0.15	5.71	1.18	10.25	0.014
POR	rs17685*	7	75,616,105	A	0.29	-4.23	-7.59	-0.87	0.014
ABCC5	rs3749442	3	183,660,585	T	0.18	-5.12	-9.29	-0.94	0.016
ABCB11	rs4148768*	2	169,887,154	T	0.09	8.39	1.50	15.28	0.017
SLC16A1	rs9429505	1	113,454,571	C	0.24	4.72	0.83	8.61	0.017
ABCB1	rs9282564	7	87,229,440	G	0.10	-6.21	-11.33	-1.08	0.018
VKORC1	rs2884737	16	31,105,554	G	0.30	3.85	0.61	7.10	0.020
MAT1A	rs9285726	10	82,035,150	T	0.35	3.81	0.56	7.05	0.021
GSTP1	rs1138272*	11	67,353,579	T	0.11	6.77	1.00	12.54	0.021
NQO1	rs10517	16	69,743,760	T	0.13	-4.91	-9.12	-0.70	0.022
ABCB11	rs3770603	2	169,883,218	A	0.09	8.09	1.14	15.03	0.023
XDH	rs2295475	2	31,589,847	T	0.32	4.60	0.55	8.65	0.026
SLC6A6	rs9036	3	14,530,721	G	0.26	3.71	0.44	6.99	0.026
ADH7	rs1573496	4	100,349,669	C	0.11	-4.57	-8.73	-0.41	0.031
CYP4F8	rs4646523	19	15,726,487	G	0.38	3.10	0.27	5.93	0.032
SLC22A12	rs11231825	11	64,360,274	T	0.34	-4.12	-7.91	-0.33	0.033
FMO3	rs2066534	1	171,077,372	G	0.16	-4.05	-7.84	-0.26	0.036
ADH7	rs971074	4	100,341,861	A	0.12	-4.28	-8.30	-0.26	0.037
CYP1B1	rs1800440	2	38,298,139	G	0.14	-5.25	-10.29	-0.20	0.042
FMO2	rs2307492	1	171,168,545	C	0.07	-3.63	-7.16	-0.09	0.044
SPN	rs4788172	16	29,668,253	A	0.06	-5.08	-10.07	-0.09	0.046
POR	rs6965343	7	75,592,925	T	0.38	-3.21	-6.36	-0.05	0.046

MTX, methotrexate; SNP, single nucleotide polymorphism; Chr, chromosome; MAF, minor allele frequency; 95% CI, 95% confidence interval; P, p-value.

Covariate included in statistical model is age.

Effect sizes and 95% CI are reported for the minor allele.

A coefficient below 0 indicates that carriers of minor allele have lower creatinine plasma levels compared to carriers of the major allele.

The following variants are in linkage disequilibrium (r^2>0.80): rs2230028 (*ABCB4*) and rs7785206 (*CROT*), rs4715332 (*GSTA1*) and rs4715354 (*GSTA5*), all depicted variants within *ABCB11*, *ADH7* and *VKORC1* (except rs7294 and rs2884737).

* variant is also associated with MTX plasma levels at 48 hours (P<0.05, Table S2).

Table S4. Genetic variants associated with creatinine plasma levels, 1 week after MTX infusion (P<0.05)

Gene	SNP	Chr	Position	Minor allele	MAF	Coefficient	95% CI		P
CYP1B1	rs1800440	2	38,298,139	G	0.14	-8.66	-13.07	-4.26	0.0001
CYP3A43	rs17342647	7	99,459,256	T	0.08	8.06	3.52	12.59	0.0005
SLC22A3	rs668871	6	160,769,811	T	0.47	-5.03	-8.56	-1.51	0.005
SLCO3A1	rs3743369	15	92,707,569	G	0.42	-4.40	-7.57	-1.23	0.006
SLC13A1	rs1880179	7	122,838,633	A	0.41	-3.44	-6.03	-0.86	0.009
SLC22A2	rs624249*	6	160,679,400	T	0.42	-4.24	-7.48	-0.99	0.011
SLCO5A1	rs16936279	8	70,584,809	C	0.12	-5.38	-9.61	-1.14	0.013
SLCO3A1	rs1517618	15	92,647,645	G	0.17	-5.12	-9.17	-1.06	0.013
CDA	rs2072671	1	20,915,701	C	0.33	-4.47	-8.03	-0.91	0.014
SLC13A1	rs2204295	7	122,838,090	G	0.40	-3.22	-5.81	-0.63	0.015
ATP7B	rs2277448	13	52,585,548	C	0.37	-4.30	-7.84	-0.77	0.017
SLC22A11	rs2078267	11	64,334,114	C	0.47	-4.30	-7.93	-0.68	0.020
CYP39A1	rs2277119	6	46,609,905	A	0.25	4.89	0.63	9.14	0.024
FMO2	rs2307492	1	171,168,545	C	0.07	-4.93	-9.24	-0.62	0.025
ATP7B	rs1051332	13	52,507,720	A	0.46	4.27	0.50	8.03	0.026
UGT2B7	rs7668258	4	69,962,078	C	0.42	3.52	0.39	6.65	0.028
UGT2B7	rs7662029	4	69,961,912	G	0.42	3.52	0.39	6.65	0.028
UGT2B7	rs7439366	4	69,964,338	C	0.42	3.48	0.35	6.61	0.029
UGT2B7	rs7438284	4	69,964,337	T	0.42	3.48	0.35	6.61	0.029
GSTP1	rs1138272*	11	67,353,579	T	0.11	6.15	0.58	11.71	0.030
VKORC1	rs7294	16	31,102,321	A	0.33	-3.34	-6.46	-0.22	0.036
CYP4A11	rs11211402	1	47,392,054	G	0.16	-4.20	-8.17	-0.23	0.038
VKORC1	rs2884737	16	31,105,554	G	0.30	3.57	0.19	6.95	0.039
SLC22A12	rs11231825	11	64,360,274	T	0.34	-3.77	-7.35	-0.19	0.039
VKORC1	rs8050894	16	31,104,509	C	0.48	3.40	0.17	6.62	0.039
CYP2A6	rs28399433	19	41,356,379	G	0.07	6.52	0.33	12.70	0.039
CROT	rs31652	7	87,029,687	G	0.15	-4.31	-8.49	-0.13	0.043
VKORC1	rs9934438	16	31,104,878	T	0.47	3.36	0.10	6.61	0.043
CYP4F12	rs609290	19	15,789,140	A	0.07	5.73	0.13	11.33	0.045
CYP4F12	rs609636	19	15,789,098	A	0.07	5.73	0.13	11.33	0.045
ABCC4	rs4148551	13	95,673,518	G	0.42	-3.42	-6.79	-0.05	0.047
ABCC4	rs3742106	13	95,673,791	G	0.42	-3.48	-6.93	-0.04	0.047
ABCC1	rs8187858	16	16,162,039	T	0.09	-4.68	-9.32	-0.03	0.048

MTX, methotrexate; SNP, single nucleotide polymorphism; Chr, chromosome; MAF, minor allele frequency; 95% CI, 95% confidence interval; P, p-value.

Covariates included in statistical model are age and MTX cumulative dose.

Effect sizes and 95% CI are reported for the minor allele.

A coefficient below 0 indicates that carriers of minor allele have lower creatinine plasma levels compared to carriers of the major allele.

The following variants are in linkage disequilibrium (r^2>0.80): all depicted variants within *ABCC4, CYP4F12, SLC3A1, UGT2B7* and *VKORC1* (except rs7294 and rs2884737).

* variant is also associated with MTX plasma levels at 48 hours (P<0.05, Table S2).

Table S5. Genetic variants associated with ALAT, 1 week after MTX infusion (P<0.05)

Gene	SNP	Chr	Position	Minor allele	MAF	Linear multivariate GEE analysis with ALAT levels				Logistic multivariate GEE analysis of CTCAE v5 ALAT increase, grade 0-1 compared to grade 2-4			
						**Coefficient**	**95% CI**		**P**	**Coefficient**	**95% CI**		**P**
CYP4F2	rs3093153	19	16,001,215	T	0.05	0.67	0.32	1.03	0.0002	2.32	0.91	3.73	0.001
PPARD	rs6906237	6	35,375,526	A	0.05	0.78	0.34	1.22	0.0005	1.24	0.12	2.37	0.030
CYP2E1	rs2070673	10	135,340,567	A	0.15	-0.44	-0.71	-0.18	0.001	-0.91	-1.67	-0.14	0.020
CYP2E1	rs2515641	10	135,351,362	T	0.09	-0.65	-1.04	-0.25	0.001	-1.43	-2.50	-0.35	0.009
PTGIS	rs5629	20	48,129,706	A	0.24	-0.38	-0.62	-0.15	0.002	-0.67	-1.19	-0.14	0.012
NAT1	rs4986993	8	18,080,747	T	0.27	-0.38	-0.61	-0.14	0.002	-0.66	-1.18	-0.13	0.015
CYP2A6	rs1137115	19	41,356,281	A	0.33	-0.30	-0.50	-0.10	0.004	-0.67	-1.17	-0.16	0.009
PPARD	rs6457816	6	35,362,848	C	0.07	0.64	0.19	1.08	0.005	1.00	-0.01	2.01	0.053
PPARD	rs6922548*	6	35,353,523	G	0.06	0.68	0.20	1.15	0.005	1.09	-0.02	2.20	0.055
PPARD	rs7739752*	6	35,339,035	T	0.06	0.68	0.20	1.15	0.005	1.09	-0.02	2.20	0.055
CYP1B1	rs1056836*	2	38,298,203	G	0.42	-0.29	-0.50	-0.08	0.006	-0.43	-0.95	0.08	0.101
SLCO1B1	rs11045819	12	21,329,813	A	0.25	0.34	0.09	0.59	0.007	0.58	-0.16	1.32	0.125
SLCO1B1	rs4149056	12	21,331,549	C	0.16	-0.43	-0.75	-0.12	0.007	-0.75	-1.56	0.05	0.067
CBR1	rs2835265	21	37,444,696	T	0.10	0.51	0.14	0.89	0.007	1.25	0.11	2.38	0.032
CYP1B1	rs1056837*	2	38,298,150	T	0.42	-0.28	-0.49	-0.07	0.009	-0.43	-0.94	0.09	0.106
CYP2D6	rs3892097	22	42,524,947	A	0.22	0.31	0.08	0.54	0.009	1.23	0.57	1.90	0.0003
SLC7A7	rs1805059	14	23,282,449	G	0.35	-0.29	-0.50	-0.07	0.010	-0.66	-1.09	-0.23	0.003
ABCC4	rs2274406	13	95,858,996	A	0.27	-0.37	-0.66	-0.09	0.010	-0.80	-1.46	-0.14	0.018
ABCC4	rs2274405	13	95,858,978	A	0.26	-0.37	-0.66	-0.09	0.010	-0.80	-1.46	-0.14	0.018
CYP2B6	rs2279343	19	41,515,263	G	0.30	-0.29	-0.51	-0.07	0.011	-0.30	-0.81	0.20	0.243
GSTP1	rs1138272*	11	67,353,579	T	0.11	0.42	0.09	0.75	0.014	0.82	-0.08	1.72	0.074
SLC28A1	rs2305367	15	85,476,441	A	0.41	0.34	0.06	0.62	0.017	0.66	0.13	1.19	0.014
ABCB4	rs1202283	7	87,082,292	T	0.50	0.27	0.04	0.49	0.019	0.41	-0.16	0.98	0.160
CYP2A6	rs8192729	19	41,350,996	A	0.08	0.43	0.07	0.79	0.019	1.75	0.66	2.84	0.002
PPARD	rs6901410*	6	35,330,030	C	0.06	0.62	0.10	1.15	0.020	1.02	-0.10	2.15	0.075
ALDH2	rs886205	12	112,204,427	G	0.21	-0.34	-0.63	-0.05	0.021	-0.45	-1.07	0.16	0.146
SULT1C4	rs1402467	2	108,994,808	G	0.24	-0.30	-0.56	-0.04	0.024	-0.45	-1.04	0.13	0.131

ABP1	rs10893	7	150,555,915	G	0.34	0.24	0.03	0.46	0.025	0.63	0.07	1.19	0.027
CYP7A1	rs8192879	8	59,403,576	A	0.42	-0.32	-0.61	-0.04	0.026	-0.47	-1.15	0.21	0.172
ABCC4	rs1059751	13	95,672,950	C	0.45	0.22	0.03	0.41	0.027	0.43	-0.08	0.94	0.100
ABCC4	rs4148553	13	95,673,135	A	0.45	0.22	0.02	0.41	0.029	0.42	-0.08	0.93	0.100
SLCO1B1	rs4149057	12	21,331,599	T	0.39	-0.26	-0.50	-0.02	0.034	-0.41	-0.96	0.15	0.152
SLC6A6	rs9036	3	14,530,721	G	0.26	0.22	0.01	0.42	0.035	0.62	0.06	1.18	0.029
SLCO5A1	rs1138541	8	70,584,628	A	0.26	0.27	0.02	0.52	0.036	0.34	-0.25	0.93	0.256
CHST2	rs3755739	3	142,837,272	A	0.24	-0.28	-0.54	-0.02	0.036	-0.77	-1.37	-0.17	0.012
CYP7A1	rs13251066	8	59,417,753	C	0.44	-0.30	-0.58	-0.01	0.039	-0.48	-1.14	0.17	0.147
NR1I2	rs3814055	3	119,500,035	T	0.35	-0.22	-0.43	-0.01	0.042	-0.26	-0.76	0.24	0.304
SLC22A4	rs1050152	5	131,676,320	T	0.42	0.23	0.01	0.45	0.042	0.66	0.19	1.14	0.006
ABP1	rs1049793	7	150,557,665	G	0.34	0.22	0.01	0.44	0.043	0.54	-0.01	1.09	0.052
ABP1	rs12179	7	150,557,622	A	0.34	0.22	0.01	0.44	0.043	0.54	-0.01	1.09	0.052
PON3	rs13226149	7	95,025,600	T	0.30	0.23	0.01	0.45	0.044	0.59	-0.04	1.21	0.066
ABCC4	rs4148551	13	95,673,518	G	0.42	-0.23	-0.45	0.00	0.046	-0.44	-0.98	0.10	0.111
SLC28A3	rs7853758	9	86,900,926	T	0.10	-0.49	-0.97	-0.01	0.046	-1.11	-1.97	-0.25	0.011
HMGCR	rs5909	5	74,656,175	A	0.08	0.46	0.01	0.92	0.047	0.67	-0.44	1.77	0.239
SLC7A7	rs1061040	14	23,242,828	A	0.10	-0.53	-1.05	-0.01	0.047	-0.91	-1.89	0.08	0.070
GSTM2	rs592792	1	110,211,956	T	0.19	0.35	0.00	0.69	0.047	0.76	-0.04	1.55	0.063
POR	rs17685*	7	75,616,105	A	0.29	-0.27	-0.54	0.00	0.049	-1.01	-1.71	-0.32	0.004
XDH	rs2295475	2	31,589,847	T	0.32	0.27	0.00	0.54	0.049	0.57	-0.11	1.25	0.101
ABCB4	rs2097937*	7	87,030,903	C	0.24	-0.28	-0.56	0.00	0.049	-0.44	-1.03	0.14	0.138

ALAT, alanine aminotransferase; MTX, methotrexate; SNP, single nucleotide polymorphism; Chr, chromosome; MAF, minor allele frequency; 95% CI, 95% confidence interval; P, p-value.

Covariates included in the linear and in the logistic GEE models are age and creatinine plasma levels at week 1.

Effect sizes and 95% CI are reported for the minor allele.

A coefficient below 0 indicates that carriers of minor allele have lower ALAT levels compared to carriers of the major allele.

The following variants are in linkage disequilibrium (r^2>0.80): rs2274406 and rs2274405 (*ABCC4*), rs1059751 and rs4148553 (*ABCC4*), all depicted variants within *ABP1*, *CYP1B1*, *CYP7A1*, *PPARD* (except rs6906237, which is linkage disequilibrium with rs7739752).

* variant is also associated with MTX plasma levels at 48 hours (P<0.05, Table S2).

Table S6: Genetic variants associated with ASAT, 1 week after MTX infusion (P<0.05)

Gene	SNP	Chr	Position	Minor allele	MAF	Linear multivariate GEE analysis with ASAT levels				Logistic multivariate GEE analysis of CTCAE v5 ASAT increase, grade 0-1 compared to grade 2-4			
						**Coefficient**	**95% CI**		**P**	**Coefficient**	**95% CI**		**P**
ALDH2	rs886205	12	112,204,427	G	0.21	-0.43	-0.67	-0.20	0.0003	-0.53	-1.09	0.03	0.062
PTGIS	rs5629	20	48,129,706	A	0.24	-0.38	-0.60	-0.17	0.0004	-0.72	-1.20	-0.24	0.003
CYP4B1	rs2297810	1	47,280,859	A	0.11	-0.45	-0.70	-0.20	0.0005	-0.97	-1.68	-0.26	0.008
CYP2E1	rs2070673	10	135,340,567	A	0.15	-0.39	-0.63	-0.15	0.001	-1.01	-1.69	-0.33	0.004
CYP2A6	rs1137115	19	41,356,281	A	0.33	-0.26	-0.43	-0.09	0.002	-0.44	-0.88	0.00	0.050
CYP2B6	rs2279343	19	41,515,263	G	0.30	-0.30	-0.50	-0.10	0.003	-0.43	-0.87	0.02	0.059
GSTP1	rs1138272	11	67,353,579	T	0.11	0.44	0.14	0.75	0.004	0.81	0.12	1.49	0.021
FMO3	rs1800822	1	171,076,935	T	0.05	-0.54	-0.92	-0.15	0.006	-1.10	-2.02	-0.18	0.019
CYP1A2	rs35694136	15	75,039,613	delT	0.10	-0.32	-0.55	-0.09	0.006	-0.80	-1.41	-0.19	0.010
CYP4B1	rs4646491	1	47,280,884	T	0.10	-0.43	-0.75	-0.12	0.007	-0.93	-1.73	-0.14	0.021
CYP4B1	rs3215983	1	47,280,747	delAT	0.10	-0.43	-0.75	-0.12	0.007	-0.93	-1.73	-0.14	0.021
GSTM2	rs530021	1	110,210,780	C	0.06	-0.53	-0.92	-0.14	0.008	-1.33	-2.55	-0.11	0.033
CA5P	rs1364182	16	29,656,093	T	0.07	-0.49	-0.86	-0.12	0.009	-1.71	-3.09	-0.34	0.014
UGT2B15	rs3100	4	69,512,655	C	0.32	-0.22	-0.39	-0.06	0.009	-0.51	-0.87	-0.14	0.007
UGT1A7	rs7586110	2	234,590,527	G	0.35	0.25	0.06	0.45	0.010	0.65	0.27	1.04	0.001
NAT1	rs4986993	8	18,080,747	T	0.27	-0.30	-0.53	-0.07	0.011	-0.72	-1.21	-0.23	0.004
ABCC4	rs4148551	13	95,673,518	G	0.42	-0.21	-0.39	-0.04	0.014	-0.43	-0.81	-0.04	0.030
ABCB11	rs4148768	2	169,887,154	T	0.09	0.37	0.07	0.68	0.015	0.83	0.10	1.57	0.026
CYP4F11	rs2305801	19	16,045,141	A	0.20	0.24	0.04	0.44	0.017	0.31	-0.12	0.74	0.152
SLCO4A1	rs3787537	20	61,303,742	T	0.24	0.30	0.05	0.55	0.020	0.95	0.42	1.47	0.0004
CYP1B1	rs1056836	2	38,298,203	G	0.42	-0.23	-0.43	-0.04	0.021	-0.45	-0.86	-0.03	0.034
ABP1	rs10893	7	150,555,915	G	0.34	0.22	0.03	0.40	0.021	0.52	0.11	0.93	0.014
SLC22A4	rs1050152	5	131,676,320	T	0.42	0.22	0.03	0.41	0.021	0.58	0.17	0.99	0.005
RALBP1	rs10898	18	9,536,249	C	0.31	-0.26	-0.48	-0.04	0.022	-0.49	-0.98	-0.01	0.045
CYP2A6	rs4803381	19	41,357,344	A	0.40	-0.20	-0.37	-0.03	0.023	-0.34	-0.75	0.07	0.107
UGT2B7	rs7439366	4	69,964,338	C	0.42	-0.24	-0.45	-0.03	0.023	-0.49	-0.97	0.00	0.048
UGT2B7	rs7438284	4	69,964,337	T	0.42	-0.24	-0.45	-0.03	0.023	-0.49	-0.97	0.00	0.048
UGT2B7	rs7668258	4	69,962,078	C	0.42	-0.24	-0.45	-0.03	0.023	-0.49	-0.97	0.00	0.048

UGT2B7	rs7662029	4	69,961,912	G	0.42	-0.24	-0.45	-0.03	0.023	-0.49	-0.97	0.00	0.048
ABCC4	rs3742106	13	95,673,791	G	0.42	-0.20	-0.37	-0.03	0.025	-0.42	-0.80	-0.03	0.033
ABCB4	rs2230028	7	87,056,176	G	0.11	-0.42	-0.78	-0.05	0.025	-0.33	-1.08	0.41	0.383
SLC16A1	rs9429505	1	113,454,571	C	0.24	0.27	0.03	0.51	0.027	0.50	0.04	0.97	0.034
CYP1B1	rs1056837	2	38,298,150	T	0.42	-0.22	-0.42	-0.02	0.028	-0.44	-0.85	-0.02	0.040
SLC22A3	rs2292334	6	160,858,188	A	0.39	-0.24	-0.46	-0.03	0.028	-0.52	-1.02	-0.03	0.039
SLC10A1	rs4646285	14	70,263,648	A	0.09	-0.26	-0.50	-0.03	0.029	-0.43	-0.95	0.09	0.107
CYP2E1	rs2515641	10	135,351,362	T	0.09	-0.42	-0.81	-0.03	0.033	-0.92	-1.89	0.05	0.064
CROT	rs7785206	7	87,021,023	C	0.10	-0.43	-0.83	-0.03	0.033	-0.39	-1.21	0.42	0.341
CYP2B6	rs3745274	19	41,512,841	T	0.26	-0.24	-0.46	-0.02	0.033	-0.29	-0.75	0.17	0.215
SLCO1B1	rs11045819	12	21,329,813	A	0.25	0.24	0.02	0.45	0.035	0.29	-0.21	0.79	0.252
ABP1	rs1049793	7	150,557,665	G	0.34	0.20	0.01	0.38	0.037	0.47	0.06	0.88	0.023
ABP1	rs12179	7	150,557,622	A	0.34	0.20	0.01	0.38	0.037	0.47	0.06	0.88	0.023
CYP24A1	rs2762934	20	52,771,261	T	0.15	-0.21	-0.41	-0.01	0.039	-0.47	-0.95	0.00	0.052
CYP4F11	rs1060463	19	16,025,176	G	0.42	0.22	0.01	0.43	0.039	0.29	-0.13	0.71	0.174
PON3	rs13226149	7	95,025,600	T	0.30	0.22	0.01	0.43	0.039	0.55	0.10	1.00	0.018
GSTM2	rs592792	1	110,211,956	T	0.19	0.29	0.01	0.58	0.040	0.87	0.26	1.48	0.005
UGT2B15	rs4148269	4	69,512,847	A	0.33	-0.18	-0.36	-0.01	0.040	-0.33	-0.69	0.02	0.068
ABCC4	rs1059751	13	95,672,950	C	0.45	0.18	0.00	0.35	0.044	0.45	0.07	0.83	0.021
ABCC4	rs4148553	13	95,673,135	A	0.45	0.17	0.00	0.34	0.047	0.44	0.06	0.82	0.023
ABCB4	rs2097937	7	87,030,903	C	0.24	-0.25	-0.49	0.00	0.048	-0.02	-0.49	0.44	0.922
UGT1A1	rs3755319	2	234,667,582	C	0.40	0.20	0.00	0.41	0.050	0.56	0.14	0.97	0.008

ASAT, aspartate aminotransferase; MTX, methotrexate; SNP, single nucleotide polymorphism; Chr, chromosome; MAF, minor allele frequency; 95% CI, 95% confidence interval; P, p-value; del, deletion.

Covariates included in the linear and in the logistic GEE models are age and creatinine plasma levels at week 1.

Effect sizes and 95% CI are reported for the minor allele.

A coefficient below 0 indicates that carriers of minor allele have lower ASAT levels compared to carriers of the major allele.

The following variants are in linkage disequilibrium (r^2>0.80): rs2230028 (*ABCB4*) and rs7785206 (*CROT*), all depicted variants within *CYP4B1*, *CYP1B1*, *UGT2B15*, *UGT2B7* and *ABP1*.

* variant is also associated with MTX plasma levels at 48 hours (P<0.05, Table S2).

Table S7: Genetic variants associated with hemoglobin, 1 week after MTX infusion (P<0.05)

Gene	SNP	Chr	Position	Minor allele	MAF	Linear multivariate GEE analysis with hemoglobin levels				Logistic multivariate GEE analysis of CTCAE v5 anemia grade 0-1 compared to grade 2-4			
						**Coefficient**	**95% CI**		**P**	**Coefficient**	**95% CI**		**P**
CYP2C8	rs1058930	10	96,818,119	G	0.06	-0.37	-0.57	-0.18	0.0002	0.77	0.36	1.19	0.0003
SLC22A1	rs683369	6	160,551,204	G	0.23	-0.20	-0.31	-0.08	0.0008	0.38	0.13	0.63	0.003
CYP2C19	rs4244285	10	96,541,616	A	0.15	-0.25	-0.40	-0.10	0.0008	0.54	0.22	0.86	0.001
CYP2C18	rs2281891	10	96,493,058	T	0.15	-0.25	-0.39	-0.10	0.001	0.54	0.22	0.86	0.001
ABCB11	rs4148768	2	169,887,154	T	0.09	-0.28	-0.46	-0.10	0.003	0.61	0.24	0.98	0.001
SLC6A6	rs2341970	3	14,474,225	T	0.25	0.20	0.05	0.35	0.008	-0.33	-0.68	0.03	0.076
CHST3	rs4148944	10	73,769,543	A	0.11	-0.22	-0.38	-0.05	0.010	0.52	0.16	0.88	0.005
SLC22A1	rs628031	6	160,560,845	A	0.45	-0.13	-0.23	-0.03	0.010	0.24	-0.04	0.52	0.090
CYP1A2	rs762551	15	75,041,917	C	0.23	0.16	0.04	0.28	0.012	-0.27	-0.60	0.06	0.106
QPRT	rs3862476	16	29,687,360	C	0.07	0.28	0.06	0.50	0.012	-0.83	-1.41	-0.26	0.004
ABCB11	rs3770603	2	169,883,218	A	0.09	-0.24	-0.43	-0.04	0.017	0.47	0.09	0.86	0.015
FMO2	rs2307492	1	171,168,545	C	0.07	0.22	0.04	0.40	0.017	-0.38	-0.98	0.23	0.221
CYP3A43	rs17342647	7	99,459,256	T	0.08	-0.17	-0.32	-0.03	0.017	0.48	0.07	0.89	0.021
SLC10A2	rs279941	13	103,698,168	T	0.13	-0.20	-0.36	-0.03	0.017	0.38	-0.05	0.80	0.084
AOX1	rs16834034	2	201,542,014	G	0.09	-0.16	-0.29	-0.03	0.018	0.28	-0.01	0.57	0.057
CYP4B1	rs4646491	1	47,280,884	T	0.10	0.23	0.04	0.42	0.018	-0.67	-1.24	-0.09	0.023
CYP4B1	rs3215983	1	47,280,747	delAT	0.10	0.23	0.04	0.42	0.018	-0.67	-1.24	-0.09	0.023
CYP11B1	rs7003319	8	143,954,747	A	0.42	0.12	0.02	0.23	0.019	-0.24	-0.52	0.04	0.089
CYP11B1	rs1134095	8	143,954,290	C	0.42	0.12	0.02	0.23	0.019	-0.24	-0.52	0.04	0.089
CYP11B1	rs4736312	8	143,953,937	T	0.42	0.12	0.02	0.23	0.019	-0.24	-0.52	0.04	0.089
ADH4	rs1126672	4	100,047,812	T	0.26	0.17	0.03	0.32	0.019	-0.33	-0.68	0.03	0.069
CYP1B1	rs1056837	2	38,298,150	T	0.42	0.14	0.02	0.25	0.021	-0.25	-0.56	0.05	0.104
QPRT	rs13331798	16	29,747,291	G	0.21	0.16	0.02	0.29	0.022	-0.45	-0.80	-0.10	0.012
CYP11B1	rs5303	8	143,955,095	T	0.42	0.12	0.02	0.23	0.023	-0.23	-0.52	0.05	0.102
SLC10A2	rs188096	13	103,705,044	T	0.13	-0.19	-0.36	-0.03	0.024	0.38	-0.05	0.81	0.084
CHST3	rs4148945	10	73,769,590	T	0.49	0.13	0.01	0.25	0.028	-0.32	-0.62	-0.03	0.032
SLC15A1	rs1339067	13	99,356,612	T	0.39	-0.12	-0.22	-0.01	0.029	0.41	0.13	0.68	0.004
CYP2A13	rs1709082	19	41,601,609	G	0.07	-0.19	-0.37	-0.02	0.030	0.45	0.02	0.88	0.040

VKORC1	rs2884737	16	31,105,554	G	0.30	0.15	0.01	0.29	0.030	-0.22	-0.55	0.10	0.181
SLC7A7	rs2281677	14	23,284,572	T	0.39	-0.12	-0.24	-0.01	0.030	0.27	-0.01	0.55	0.057
CHST3	rs1871450	10	73,772,014	A	0.49	0.13	0.01	0.25	0.035	-0.32	-0.62	-0.02	0.037
CHST3	rs4148950	10	73,771,706	A	0.49	0.13	0.01	0.25	0.035	-0.32	-0.62	-0.02	0.037
CYP1B1	rs1056836	2	38,298,203	G	0.42	0.12	0.01	0.24	0.037	-0.23	-0.54	0.07	0.134
SLCO1B1	rs11045819	12	21,329,813	A	0.25	0.14	0.01	0.27	0.038	-0.39	-0.72	-0.05	0.024
SLC7A7	rs1805059	14	23,282,449	G	0.35	-0.13	-0.25	-0.01	0.038	0.31	0.02	0.60	0.038
ADH4	rs1126670	4	100,052,733	G	0.28	0.15	0.01	0.29	0.039	-0.28	-0.61	0.05	0.101
ADH4	rs1126671	4	100,048,414	A	0.28	0.15	0.01	0.29	0.039	-0.28	-0.61	0.05	0.101
SLC7A7	rs8018462	14	23,282,110	T	0.44	-0.12	-0.23	-0.01	0.040	0.26	-0.02	0.53	0.068
GSTO1	rs4925	10	106,022,789	A	0.33	-0.12	-0.24	-0.01	0.040	0.33	0.04	0.62	0.024
CYP26A1	rs10882140	10	94,842,213	G	0.45	0.11	0.01	0.22	0.040	-0.20	-0.49	0.09	0.181
SULT1E1	rs3736599	4	70,725,821	A	0.08	0.17	0.00	0.33	0.043	-0.53	-0.95	-0.11	0.014
GSTM2	rs530021	1	110,210,780	C	0.06	0.23	0.01	0.46	0.043	-0.84	-1.62	-0.06	0.034
CHST3	rs730720	10	73,772,762	T	0.50	0.12	0.00	0.24	0.046	-0.29	-0.58	0.00	0.053
CHST3	rs731027	10	73,772,336	C	0.50	0.12	0.00	0.24	0.046	-0.29	-0.58	0.00	0.053
CHST3	rs4148943	10	73,769,507	T	0.50	0.12	0.00	0.24	0.046	-0.29	-0.58	0.00	0.053
ABCB1	rs2214102	7	87,229,501	A	0.11	-0.16	-0.31	0.00	0.047	0.17	-0.26	0.60	0.433
GSTP1	rs1695	11	67,352,689	G	0.36	0.12	0.00	0.23	0.049	-0.17	-0.49	0.14	0.274

MTX, methotrexate; SNP, single nucleotide polymorphism; Chr, chromosome; MAF, minor allele frequency; 95% CI, 95% confidence interval; P, p-value; del, deletion.

Covariates included in the linear GEE model are sex, MTX cumulative and creatinine plasma levels at week 1. Covariates included in the logistic GEE model are sex, age and creatinine plasma levels at week 1.

Effect sizes and 95% CI are reported for the minor allele.

A coefficient below 0 indicates that carriers of minor allele have lower hemoglobin levels compared to carriers of the major allele.

The following variants are in linkage disequilibrium (r^2>0.80): all depicted variants within *CYP4B1*, *ABCB11*, *CYP1B1*, *ADH4*, *CYP11B1*, *CHST3* (except rs4148944), *SLC10A2* and *SLC7A7* (except rs8018462).

* variant is also associated with MTX plasma levels at 48 hours (P<0.05, Table S2).

Table S8: Genetic variants associated with thrombocyte counts, 1 week after MTX infusion (P<0.05)

Gene	SNP	Chr	Position	Minor allele	MAF	Linear multivariate GEE analysis with thrombocyte counts				Logistic multivariate GEE analysis of CTCAE v5 platelet count decrease, grade 0 compared to grade 1-4			
						**Coefficient**	**95% CI**		**P**	**Coefficient**	**95% CI**		**P**
CYP2B6	**rs4803418**	**19**	**41,511,803**	**G**	**0.31**	**-0.19**	**-0.27**	**-0.10**	**0.00003**	**0.32**	**0.01**	**0.63**	**0.046**
CYP4F8	**rs4808326**	**19**	**15,726,147**	**A**	**0.10**	**0.19**	**0.10**	**0.29**	**0.00006**	**-0.97**	**-1.55**	**-0.39**	**0.001**
CYP2B6	**rs4803419**	**19**	**41,512,792**	**T**	**0.32**	**-0.19**	**-0.28**	**-0.09**	**0.00009**	**0.32**	**0.00**	**0.65**	**0.051**
SLC13A1	rs6962039	7	122,754,358	T	0.24	-0.18	-0.29	-0.07	0.001	0.59	0.22	0.95	0.002
FMO2	rs2020870	1	171,154,959	G	0.08	0.23	0.09	0.37	0.001	-0.74	-1.31	-0.17	0.011
CYP2B6	rs2279343	19	41,515,263	G	0.30	0.16	0.05	0.27	0.003	-0.28	-0.67	0.11	0.165
SLC7A8	rs2268877	14	23,636,757	C	0.22	-0.17	-0.29	-0.06	0.004	0.49	0.08	0.89	0.018
NR1I2	rs2276707	3	119,534,153	T	0.18	0.15	0.05	0.26	0.004	-0.64	-1.10	-0.17	0.007
SLC16A1	rs9429505	1	113,454,571	C	0.24	-0.17	-0.29	-0.05	0.005	0.44	0.06	0.81	0.023
CYP4F11	rs1060463	19	16,025,176	G	0.42	-0.13	-0.22	-0.04	0.006	0.46	0.12	0.81	0.008
CYP4F11	rs3765070	19	16,040,292	T	0.41	-0.13	-0.22	-0.04	0.007	0.45	0.11	0.79	0.010
ADH4	rs1126672	4	100,047,812	T	0.26	0.16	0.04	0.27	0.007	-0.47	-0.86	-0.07	0.020
FMO3	rs1736557	1	171,080,080	A	0.10	0.16	0.04	0.28	0.008	-0.46	-0.98	0.05	0.078
SULT2B1	rs2302948	19	49,096,065	T	0.23	-0.19	-0.33	-0.05	0.008	0.69	0.23	1.15	0.003
UGT1A9	rs3832043	2	234,580,463	T	0.38	0.16	0.04	0.29	0.009	-0.44	-0.81	-0.06	0.022
SLC28A1	rs2305367	15	85,476,441	A	0.41	-0.14	-0.24	-0.03	0.009	0.42	0.08	0.77	0.016
ABCC5	rs939336	3	183,685,534	T	0.39	-0.11	-0.19	-0.02	0.012	0.24	-0.07	0.55	0.130
CYP2B6	rs8192719	19	41,518,773	T	0.25	0.14	0.03	0.25	0.015	-0.20	-0.59	0.19	0.317
CYP2B6	rs3745274	19	41,512,841	T	0.26	0.14	0.03	0.25	0.015	-0.23	-0.62	0.17	0.267
ADH1A	rs6811453	4	100,194,977	T	0.38	0.13	0.02	0.23	0.015	-0.31	-0.67	0.05	0.087
ADH1A	rs1826909	4	100,217,743	A	0.38	0.13	0.02	0.23	0.017	-0.31	-0.66	0.05	0.091
ABP1	rs12539	7	150,558,366	T	0.21	0.14	0.02	0.26	0.018	-0.38	-0.80	0.05	0.083
ADH4	rs1126670	4	100,052,733	G	0.28	0.14	0.02	0.25	0.019	-0.37	-0.77	0.03	0.070
ADH4	rs1126671	4	100,048,414	A	0.28	0.14	0.02	0.25	0.019	-0.37	-0.77	0.03	0.070
CYP4F11	rs2305801	19	16,045,141	A	0.20	-0.13	-0.23	-0.02	0.020	0.42	0.04	0.80	0.029
CYP4F11	rs8104361	19	16,034,714	T	0.20	-0.13	-0.25	-0.02	0.022	0.37	-0.03	0.78	0.068
CYP2C19	rs12248560	10	96,521,657	T	0.27	0.12	0.02	0.21	0.022	-0.30	-0.67	0.08	0.119
ALB	rs3756067	4	74,269,645	A	0.39	-0.12	-0.22	-0.02	0.024	0.58	0.23	0.93	0.001

ABCC5	rs7636910	3	183,699,516	G	0.42	0.11	0.01	0.20	0.025	-0.18	-0.50	0.14	0.274
GSTM2	rs530021	1	110,210,780	C	0.06	0.18	0.02	0.34	0.025	-0.98	-1.75	-0.21	0.013
SLC5A6	rs1395	2	27,424,636	C	0.32	0.11	0.01	0.20	0.027	-0.22	-0.55	0.11	0.192
FMO2	rs2020863	1	171,174,531	G	0.07	-0.22	-0.43	-0.02	0.031	0.48	-0.26	1.22	0.203
ADH5	rs1154400	4	100,010,010	G	0.29	0.12	0.01	0.23	0.034	-0.38	-0.74	-0.02	0.036
CYP2F1	rs58285195	19	41,622,205	C	0.05	-0.27	-0.53	-0.02	0.035	0.91	0.11	1.72	0.025
SLC7A8	rs1884545	14	23,652,004	T	0.10	-0.16	-0.32	-0.01	0.036	0.32	-0.26	0.90	0.276
CYP1B1	rs1056837	2	38,298,150	T	0.42	0.11	0.01	0.22	0.038	-0.16	-0.51	0.19	0.378
FMO2	rs2020869	1	171,178,152	G	0.12	0.15	0.01	0.30	0.040	-0.48	-1.03	0.06	0.084
HNMT	rs4646333	2	138,773,229	A	0.22	-0.13	-0.25	-0.01	0.040	0.32	-0.08	0.73	0.120
HNMT	rs1050891	2	138,771,760	G	0.22	-0.13	-0.25	-0.01	0.040	0.32	-0.08	0.73	0.120
CYP19A1	rs10046	15	51,502,986	C	0.49	-0.12	-0.24	-0.01	0.041	0.64	0.23	1.05	0.002
SLC22A2	rs624249	6	160,679,400	T	0.42	-0.11	-0.22	0.00	0.042	0.47	0.09	0.86	0.016
ADH1A	rs12512110	4	100,195,815	A	0.08	-0.17	-0.34	-0.01	0.043	0.37	-0.26	1.00	0.250
CYP4F8	rs2056822	19	15,739,597	A	0.29	0.11	0.00	0.21	0.044	-0.34	-0.70	0.01	0.060
CYP4F8	rs4239614	19	15,740,220	C	0.29	0.11	0.00	0.21	0.044	-0.35	-0.71	0.01	0.055
HNMT	rs4245861	2	138,772,694	T	0.23	-0.13	-0.25	0.00	0.047	0.33	-0.08	0.74	0.119
UGT2B7	rs28365062	4	69,964,271	G	0.10	0.14	0.00	0.29	0.047	-0.59	-1.17	-0.01	0.045

MTX, methotrexate; SNP, single nucleotide polymorphism; Chr, chromosome; MAF, minor allele frequency; 95% CI, 95% confidence interval; P, p-value.

Covariates included in linear GEE model are sex and creatinine plasma levels at week 1. Covariate included in the logistic GEE model is creatinine plasma levels at week 1.

Effect sizes and 95% CI are reported for the minor allele.

A coefficient below 0 indicates that carriers of minor allele have lower thrombocyte counts compared to carriers of the major allele.

Variants depicted in bold surpass the Bonferroni corrected p-value threshold of 9.2×10^{-5}.

The following variants are in linkage disequilibrium (r^2>0.80): rs1126672 (*ADH4*) and rs115440 (*ADH5*), rs4803418 and rs4803419 (*CYP2B6*), rs1060463 and rs3765070 (*CYP4F11*), rs2305801 and rs8104361 (*CYP4F11*), all depicted variants within *HNMT*, *ADH1A*, *ADH4* and *CYP4F8* (except rs4808326).

* variant is also associated with MTX plasma levels at 48 hours (P<0.05, Table S2).

Table S9: Genetic variants associated with leucocyte counts, 1 week after MTX infusion (P<0.05)

Gene	SNP	Chr	Position	Minor allele	MAF	Linear multivariate GEE analysis with leukocyte counts				Logistic multivariate GEE analysis of CTCAE v5 white blood cell decrease, grade 0-1 compared to grade 2-4			
						**Coefficient**	**95% CI**		**P**	**Coefficient**	**95% CI**		**P**
CYP2B6	rs4803418	19	41,511,803	G	0.31	-0.12	-0.19	-0.06	0.0003	0.36	0.06	0.66	0.019
ALB	rs3756067	4	74,269,645	A	0.39	-0.13	-0.21	-0.06	0.0007	0.62	0.23	1.00	0.002
CYP2B6	rs4803419	19	41,512,792	T	0.32	-0.11	-0.18	-0.04	0.003	0.24	-0.07	0.55	0.133
GSTZ1	rs7972	14	77,793,237	A	0.08	0.18	0.06	0.30	0.004	-0.70	-1.30	-0.11	0.020
ABCC6	rs8058696	16	16,278,869	C	0.42	0.10	0.03	0.17	0.008	-0.38	-0.76	-0.01	0.044
CYP2F1	rs58285195	19	41,622,205	C	0.05	-0.24	-0.42	-0.06	0.009	0.97	0.39	1.56	0.001
UGT2B15	rs1902023	4	69,536,084	T	0.50	0.10	0.03	0.18	0.009	-0.38	-0.73	-0.03	0.035
NR1I2	rs3814055	3	119,500,035	T	0.35	0.09	0.02	0.16	0.009	-0.55	-0.87	-0.23	0.001
UGT2B4	rs1131878	4	70,345,904	G	0.38	-0.10	-0.17	-0.02	0.013	0.35	0.03	0.68	0.035
SLCO5A1	rs1138541	8	70,584,628	A	0.26	-0.11	-0.19	-0.02	0.014	0.46	0.11	0.81	0.009
ABCC6	rs8058694	16	16,278,863	C	0.42	0.09	0.02	0.17	0.017	-0.37	-0.74	0.00	0.052
PTGIS	rs5629	20	48,129,706	A	0.24	0.10	0.02	0.19	0.017	-0.51	-0.92	-0.11	0.013
NAT2	rs1041983	8	18,257,795	T	0.36	-0.09	-0.17	-0.01	0.031	0.44	0.08	0.80	0.015
VKORC1	rs2884737	16	31,105,554	G	0.30	0.09	0.01	0.18	0.037	-0.32	-0.67	0.02	0.069
SLC13A1	rs6962039	7	122,754,358	T	0.24	-0.10	-0.20	-0.01	0.037	0.38	0.00	0.76	0.050
ADH6	rs6830685	4	100,143,184	C	0.21	-0.10	-0.19	0.00	0.039	0.17	-0.22	0.56	0.404
GSTM3	rs1799735	1	110,280,254	delAGG	0.12	0.13	0.01	0.25	0.041	-0.57	-1.36	0.22	0.159
UGT2B4	rs13119049	4	70,346,565	A	0.31	-0.09	-0.18	0.00	0.046	0.37	0.00	0.73	0.050
SPG7	rs2292954	16	89,613,123	G	0.22	-0.08	-0.16	0.00	0.049	0.24	-0.11	0.58	0.174
HNMT	rs4245861	2	138,772,694	T	0.23	-0.09	-0.19	0.00	0.049	0.18	-0.21	0.58	0.365
UGT2B4	rs13142440	4	70,346,564	A	0.31	-0.09	-0.18	0.00	0.049	0.37	0.00	0.74	0.049

MTX, methotrexate; SNP, single nucleotide polymorphism; Chr, chromosome; MAF, minor allele frequency; 95% CI, 95% confidence interval; P, p-value; del, deletion.

Covariates included in the linear and the logistic GEE model are age and creatinine plasma levels at week 1.

Effect sizes and 95% CI are reported for the minor allele.

A coefficient below 0 indicates that carriers of minor allele have lower leukocyte counts compared to carriers of the major allele.

The following variants are in linkage disequilibrium (r^2>0.80): all depicted variants within *ABCC6, CYP2B6* and *UGT2B4* (except rs1131878).

* variant is also associated with MTX plasma levels at 48 hours (P<0.05, Table S2).

Table S10: Genetic variants associated with neutrophil counts, 1 week after MTX infusion (P<0.05)

Gene	SNP	Chr	Position	Minor allele	MAF	Linear multivariate GEE analysis with neutrophil counts				Logistic multivariate GEE analysis of CTCAE v5 neutrophil count decrease, grade 0-2 compared to grade 3-4			
						**Coefficient**	**95% CI**		**P**	**Coefficient**	**95% CI**		**P**
PTGIS	rs5629	20	48,129,706	A	0.24	0.19	0.08	0.29	0.0008	-0.63	-1.09	-0.17	0.007
UGT2B4	rs1131878	4	70,345,904	G	0.38	-0.16	-0.25	-0.07	0.0008	0.50	0.12	0.88	0.010
UGT2B4	rs13119049	4	70,346,565	A	0.31	-0.17	-0.28	-0.06	0.002	0.54	0.13	0.96	0.011
ALB	rs3756067	4	74,269,645	A	0.39	-0.17	-0.28	-0.06	0.003	0.53	0.14	0.93	0.008
UGT2B4	rs13142440	4	70,346,564	A	0.31	-0.17	-0.28	-0.06	0.003	0.52	0.10	0.94	0.015
UGT2B4	rs1966151	4	70,346,127	C	0.25	0.15	0.05	0.26	0.005	-0.41	-0.86	0.05	0.080
ADH6	rs6830685	4	100,143,184	C	0.21	-0.18	-0.31	-0.05	0.005	0.74	0.35	1.12	0.000
CYP2B6	rs4803418	19	41,511,803	G	0.31	-0.14	-0.24	-0.04	0.007	0.22	-0.14	0.57	0.227
CA5P	rs1364182	16	29,656,093	T	0.07	0.25	0.07	0.43	0.007	-0.37	-1.06	0.32	0.296
SLCO5A1	rs1138541	8	70,584,628	A	0.26	-0.17	-0.29	-0.05	0.007	0.25	-0.17	0.68	0.239
CYP2A13	rs1709082	19	41,601,609	G	0.07	-0.20	-0.35	-0.05	0.008	0.59	0.03	1.15	0.040
CYP4Z1	rs7512729	1	47,578,471	C	0.41	0.14	0.04	0.25	0.009	-0.32	-0.68	0.04	0.080
NR1I2	rs3814055	3	119,500,035	T	0.35	0.14	0.04	0.25	0.009	-0.55	-1.00	-0.09	0.019
CDA	rs2072671	1	20,915,701	C	0.33	-0.15	-0.26	-0.04	0.010	0.20	-0.17	0.57	0.288
CDA	rs2072671	1	20,915,701	C	0.33	-0.15	-0.26	-0.04	0.010	0.20	-0.17	0.57	0.288
CA5P	rs11150564	16	29,666,736	T	0.44	-0.14	-0.24	-0.03	0.012	0.56	0.17	0.96	0.005
ADH6	rs10002894	4	100,141,240	C	0.22	-0.16	-0.29	-0.03	0.015	0.74	0.35	1.13	0.0002
CYP2B6	rs4803419	19	41,512,792	T	0.32	-0.12	-0.22	-0.01	0.026	0.18	-0.18	0.53	0.327
CHST1	rs9787901	11	45,696,153	T	0.08	0.21	0.02	0.40	0.028	-0.97	-1.84	-0.10	0.029
CYP2F1	rs58285195	19	41,622,205	C	0.05	-0.32	-0.60	-0.03	0.030	0.92	0.20	1.64	0.013
UGT2B15	rs1902023	4	69,536,084	T	0.50	0.14	0.01	0.26	0.030	-0.36	-0.79	0.07	0.099
CYP2S1	rs338599	19	41,700,493	G	0.05	0.30	0.02	0.58	0.034	-0.48	-1.44	0.48	0.325
SLC22A1	rs628031	6	160,560,845	A	0.45	-0.12	-0.24	-0.01	0.038	0.22	-0.20	0.65	0.304
QPRT	rs13331798	16	29,747,291	G	0.21	0.14	0.01	0.27	0.039	-0.63	-1.12	-0.14	0.011
SLC10A1	rs4646285	14	70,263,648	A	0.09	0.22	0.01	0.42	0.042	-0.37	-1.20	0.46	0.378
CYP4F11	rs2305801	19	16,045,141	A	0.20	-0.14	-0.27	0.00	0.044	0.52	0.07	0.97	0.023
ABCC6	rs8058696	16	16,278,869	C	0.42	0.12	0.00	0.23	0.049	-0.35	-0.80	0.09	0.122
SLC28A1	rs2290272	15	85,447,431	A	0.32	-0.11	-0.23	0.00	0.049	0.35	-0.04	0.74	0.080

MTX, methotrexate; SNP, single nucleotide polymorphism; Chr, chromosome; MAF, minor allele frequency; 95% CI, 95% confidence interval; P, p-value.

Covariates included in linear GEE model are MTX cumulative dose and creatinine plasma levels at week 1. Covariate included in the logistic GEE model is creatinine plasma levels at week 1.

Effect sizes and 95% CI are reported for the minor allele.

A coefficient below 0 indicates that carriers of minor allele have lower neutrophil counts compared to carriers of the major allele.

The following variants are in linkage disequilibrium (r^2>0.80): rs13119094 and rs13142440 (*UGT2B4*), all depicted variants within *ADH6* and *CYP2B6*.

* variant is also associated with MTX plasma levels at 48 hours (P<0.05, Table S2).

Table S11: National Cancer Institute Common Terminology Criteria for Adverse Events (CTCAE) Version 5.0, used to grade laboratory toxicity markers

CTCAE Term	Grade 0	Grade 1	Grade 2	Grade 3	Grade 4	Grade 5
**Alanine aminotransferase increased**	<ULN if baseline was normal; <1.5 x baseline if baseline was abnormal	>ULN - 3.0 x ULN if baseline was normal; 1.5 - 3.0 x baseline if baseline was abnormal	>3.0 - 5.0 x ULN if baseline was normal; >3.0 - 5.0 x baseline if baseline was abnormal	>5.0 - 20.0 x ULN if baseline was normal; >5.0 - 20.0 x baseline if baseline was abnormal	>20.0 x ULN if baseline was normal; >20.0 x baseline if baseline was abnormal	-
**Aspartate aminotransferase increased**	<ULN if baseline was normal; <1.5 x baseline if baseline was abnormal	>ULN - 3.0 x ULN if baseline was normal; 1.5 - 3.0 x baseline if baseline was abnormal	>3.0 - 5.0 x ULN if baseline was normal; >3.0 - 5.0 x baseline if baseline was abnormal	>5.0 - 20.0 x ULN if baseline was normal; >5.0 - 20.0 x baseline if baseline was abnormal	>20.0 x ULN if baseline was normal; >20.0 x baseline if baseline was abnormal	-
**Anemia**	Hgb >LLN	Hgb <LLN - 10.0 g/dL; <LLN - 6.2 mmol/L; <LLN - 100 g/L	Hgb <10.0 - 8.0 g/dL; <6.2 - 4.9 mmol/L; <100 - 80 g/L	Hgb <8.0 g/dL; <4.9 mmol/L; <80 g/L; transfusion indicated	Life-threatening consequences; urgent intervention indicated	Death
**Platelet count decreased**	>LLN	<LLN - 75,000/mm3; <LLN - 75.0 x 10e9 / L	<75,000 - 50,000/mm3; <75.0 - 50.0 x 10e9 /L	<50,000 - 25,000/mm3; <50.0 - 25.0 x 10e9 /L	<25,000/mm3; <25.0 x 10e9 /L	-
**White blood cell decreased**	>LLN	<LLN - 3000/mm3; <LLN - 3.0 x 10e9 /L	<3000 - 2000/mm3; <3.0 - 2.0 x 10e9 /L	<2000 - 1000/mm3; <2.0 - 1.0 x 10e9 /L	<1000/mm3; <1.0 - 10e9 /L	-
**Neutrophil count decreased**	>LLN	<LLN - 1500/mm3; <LLN - 1.5 x 10e9 /L	<1500-1000/mm3; <1.5 - 1.0 x 10e9 /L	<1000 - 500/mm3; <1.0 - 0.5 x 10e9 /L	<500/mm3; <0.5 - 10e9 /L	-
**Creatinine increased**	<ULN	>ULN - 1.5 x ULN	>1.5 - 3.0 x baseline; >1.5 - 3.0 x ULN	>3.0 x baseline; >3.0 - 6.0 x ULN	>6.0 x ULN	-

ULN, upper limit of normal; LLN, lower limit of normal; Hgb, hemoglobin.

Grades depicted by a green cell are considered ‘controls’ in binary genetic association analyses. Grades depicted by a red cell are considered ‘cases’.
